# Comparative environmental evaluation of highway speed limit strategies: a microsimulation study

**DOI:** 10.1038/s41598-026-66537-7

**Published:** 2026-08-21

**Authors:** Usama Elrawy Shahdah, Marwa Elharoun, Eman K. Ali, Sania Reyad Elagamy

**Affiliations:** Public Works Engineering Department, Faculty of Engineering, Mansoura University, Mansoura, 35516 Egypt

**Keywords:** Speed management, Traffic emissions, SUMO micro-simulation, Elasticity analysis, Differential speed limits, Engineering, Environmental sciences, Mathematics and computing

## Abstract

Speed management policies are cost-effective tools for mitigating traffic emissions, yet comparative studies remain fragmented. This study addresses this gap through a novel framework comparing three highway speed limit strategies, Uniform Speed Limit (USL), Differential Speed Limit (DSL), and Lane-Based Speed Limit (LBSL), using microsimulation-based elasticity analysis across 432 scenarios. Contributions include: (1) the first comprehensive multi-pollutant comparison under controlled conditions; (2) a systematically compared framework demonstrating log models’ superiority for emission prediction; (3) quantified interaction effects showing elastic responses to traffic volume but inelastic responses to speed changes; and (4) a comparative framework that offers qualitative guidance for policy in contexts characterized by heterogeneous vehicle fleets. Results show LBSL significantly increases emissions relative to DSL (NO_x_ +46.8%, CO_₂_ +28%), while USL presents trade-offs (CO − 7.6%, but NO_x_ +14.7%). DSL demonstrates the most favorable relative environmental performance under the tested scenarios. Findings emphasize that effective policy must simultaneously consider traffic demand elasticity, speed regimes, and fleet composition rather than uniform approaches.

## Introduction

Although transportation systems are essential for economic progress and social operation, they globally exemplify a major contributor to environmental degradation and public health troubles. The transportation sector contributes about 24% of global carbon dioxide (CO_₂_) emissions, resulting from fuel burning, where road transport introduces about three-quarters of these emissions^1^. Not only do road vehicles emit greenhouse gases, but they also emit a complicated mix of pollutants comprising carbon monoxide (CO), nitrogen oxides (NO_x_), hydrocarbons (HC), and particulate matter (PMx), all of which negatively affect human health and air quality^2^. Based on the European Environment Agency (EEA) report, road transport remains the highest source of nitrogen oxide emissions in Europe, providing 39% of total NO_x_ emissions and reducing air quality significantly^3^.

Highway traffic with high volumes and constant speeds presents exceptional challenges for managing emissions. Despite highways enabling effective long-distance travel, they condense vehicular emissions along definite paths, impacting local atmospheric situations and air quality^4^. The correlation between traffic flow characteristics and emissions is nonlinear and complicated, affected by several factors including traffic volume, acceleration patterns, vehicle speed, traffic composition, and roadway geometry^5,6^. It is essential to understand these correlations to develop significant strategies to alleviate the impacts of transportation-related environmental issues while keeping economic production and mobility. Traffic volume is defined as the number of vehicles crossing a certain point per unit time, and it essentially affects emissions levels. Naturally, higher traffic volumes result in greater cumulative emissions, but the relationship is not basically proportional. The increase in traffic volume results in intense interactions between vehicles, affecting acceleration patterns, speeds, and the efficiency of overall traffic flow^7^. The appearance of stop-and-go traffic patterns occurs under congested conditions, described by recurrent acceleration and deceleration cycles that increase emissions rates substantially compared with free-flow conditions^8^.

Traffic elasticity of emissions is known as the emissions percentage change resulting from the traffic volume percent change, and it gives valuable insight into this relationship. In case elasticity values are higher than one, this means that emissions increase with traffic more than proportionally, whereas values less than one indicate economies of scale in emissions production. Based on previous studies, resilience estimates vary according to the specific situation, traffic conditions, and pollutants^9^. It is essential to understand these elasticities for assessing the emission effects of traffic demand management strategies and land use policies that impact travel volumes. Recent research has increasingly employed log regression specifications to estimate traffic-emission elasticities directly. This approach, in which both the dependent variable (emissions) and independent variable (traffic volume) are logarithmically transformed, yields coefficient estimates that can be interpreted directly as elasticity^10^. Such models offer advantages in handling the typically right-skewed distributions of emission data and in capturing proportional rather than absolute relationships between variables^11^.

Total emissions depend on the composition of the vehicle fleet, especially the proportion of heavy vehicles such as trucks and trailers. Heavy vehicles differ from passenger cars in their engine technology, the type of fuel used, aerodynamic characteristics, weight, and operational patterns. These differences lead to higher emission rates per kilometer travelled by vehicle for most pollutants^12^. The European Environment Agency (2022) reported that heavy vehicles account for about 27% CO₂ emissions from road transport, even though they make up only around 5% of the vehicle fleet, which points to a disproportionate environmental impact^3^.

Nitrogen oxide emissions vary strongly with the proportion of heavy vehicles. Most heavy trucks run on diesel engines, and these engines emit much higher NO_x_ than gasoline engines because they operate with higher combustion temperatures and higher compression ratios^13^. Even with advances in after-treatment systems such as selective catalytic reduction (SCR), measured NO_x_ emissions from heavy vehicles in on-road testing often exceed legal limits, especially during high-load conditions typical of highway operation^14^. This sensitivity indicates that policies aimed at reducing the proportions of heavy vehicles or adjusting how they operate could lead to substantial reductions in NO_x_ emissions. The interface between heavy vehicles and traffic flow patterns makes it harder to estimate emissions accurately. Because heavy vehicles accelerate more slowly and tend to lose speed on grades, they can slow the traffic stream, lead to platooning, and change the speed distribution of other vehicles^15^. Aerodynamic drafting, when vehicles travel closely behind trucks, can change fuel consumption patterns. These interactive effects show why emission models and policy studies should account for fleet composition directly, rather than treating the fleet as a single average vehicle.

Speed limit policies are one of the clearest and most practical traffic management tools that transportation agencies can implement. By setting and enforcing vehicle speed limits, authorities can influence not only road safety but also traffic flow patterns, fuel use, and the rate of emissions^16,17^. The impact of speed limits on emissions is not simple; it varies with the speed range, traffic conditions, and the characteristics of the vehicle fleet. Because the issue is complex, we need careful empirical analysis to guide evidence-based policy choices on the best approaches to managing speed. Many studies have examined how vehicle speed relates to emissions and fuel use, and they often report a U-shaped curve in which both low and high speeds tend to produce higher levels for most pollutants and higher fuel consumption. At low speeds, vehicles tend to run less efficiently because they often speed up and slow down, spend time idling, and may not stay in the best gear. As a result, emissions per unit of distance traveled are higher^18^. As vehicle speed rises to a moderate range, the engine tends to run more efficiently, which lowers emissions per kilometer. At higher speeds, aerodynamic drag rises sharply, which demands more power and results in higher fuel use and emissions^12^. The relationship is not linear, which suggests that some driving speeds produce the lowest emissions. The speed that minimizes emissions can differ across pollutants and depends on characteristics of the vehicle.

Empirical studies consistently report a U-shaped relationship across various contexts. Ding and Rakha reported that light-duty vehicle emissions were lowest at speeds of about 60 to 90 km/h and increased at speeds below or above this range^19^. Barth and Boriboonsomsin also found that modest reductions in freeway speeds can lower emissions, especially CO₂, and reduce fuel use^20^. In a more recent study, Huang et al. analyzed real-world driving data and found that emission factors changed with vehicle speed, with the lowest emission rates for most pollutants occurring at about 70–90 km/h^21^.

As emissions increase in a nonlinear way as speed rises, especially at higher speeds, speed limit choices can change overall emissions more than a simple linear assumption would predict. Linear models may not reflect how speed changes affect outcomes in practice, which can lead to policy recommendations that are not well aligned with the real effects. In recent studies, researchers have used quadratic and logarithmic model forms to capture these nonlinear patterns more accurately^22,23^. Choosing the right functional form is necessary to produce accurate emission forecasts and to support reliable policy analysis.

Transportation agencies set speed limits in different ways to manage trade-offs among safety, mobility, and environmental sustainability. Current work on highway speed management often groups the main strategies into three categories: Uniform Speed Limits, Differential Speed Limits, and Lane-Based Speed Limits.

*Uniform speed limits (USL)* set the same maximum speed for all vehicles, without adjusting for vehicle type or which lane a driver is in. This approach makes enforcement and compliance easier by giving all road users clear, consistent guidance^24^. USL systems are used in many jurisdictions and are familiar to most drivers. Uniform speed limits may not reflect the different operating characteristics of vehicle types, which can lead to speed differences and interaction problems between passenger vehicles and heavy trucks^25^.

*Differential speed limits (DSL)* set different maximum speeds based on vehicle type^26^. In most cases, heavy trucks are assigned a lower speed limit than passenger vehicles. The rationale for DSL is based on the longer stopping distances of heavy vehicles, their distinct handling characteristics, and the higher crash severity when trucks are involved^27^. Some researchers argue that DSL can improve safety because it separates typical speed ranges across different vehicle types. Others argue that larger speed gaps between lanes may lead to more lane changes and more points where vehicles interact, which could raise the risk of conflicts^28^. From emissions standpoint, DSL may change traffic flow patterns and, as a result, affect total emission levels, but the empirical evidence on this relationship is still limited.

*Lane-based speed limits (LBSL) *set different posted speeds for different lanes on the same roadway^29^. In most cases, the leftmost passing lane is assigned a higher limit, while the rightmost lane has a lower limit. This strategy groups vehicles by their preferred speed to better manage traffic flow, which may reduce conflicts from lane changes and improve overall traffic efficiency^30,31^. LBSL has been studied as a way to support different driver speed preferences while keeping traffic flow orderly. The implications of LBSL for vehicle emissions remain under-explored, leaving a clear gap in the current literature.

It is still unclear which of these strategies (USL, DSL, LBSL) reduce emissions more, and this needs further study. Many studies (for example^17,32–34^ look at single parts of speed management and emissions, but direct comparisons across different strategy types, multiple pollutants, and varied traffic conditions are still limited.

Despite substantial research on speed management and emissions, four critical gaps persist in literature. First, fragmented strategy comparisons: existing studies typically examine a particular approach to speed management individually, and though some research comparatively examines the effectiveness of two approaches, overall evaluations of all three approaches, namely, USL, DSL, and LBSL, under similar operating environments are absent. Second, single-pollutant focus: while most research on pollutant emissions focuses on a single gas like CO₂, the trade-offs between different pollutants with varied implications for public health and the environment are usually overlooked; moreover, the public health implications of pollutants like NO_x_ and PMx are generally given little attention in evaluations of speed strategies. Third, limited methodological framework for elasticity estimation: past research typically uses different model specifications, such as linear or polynomial functional forms, which do not yield policy-relevant elasticity estimates directly, and there is no research that has compared functional forms to suggest which model specification performs better in terms of meeting three criteria: (a) statistical performance, (b) prediction consistency, and (c) elasticity estimate significance. Fourth, developed-country context bias: existing evidence derives predominantly from developed nations with modern vehicle fleets, strong enforcement capacity, and specific traffic patterns, meaning that applicability to developing countries characterized by heterogeneous vehicle fleets, variable enforcement, and different road user behaviors remain unverified.

This study addresses these gaps through three novel contributions. First, in terms of methodological innovation, it develops and systematically evaluates an integrated microsimulation-elasticity framework for the first time by systematically comparing various functional forms (linear, quadratic, and logarithmic) for predicting emissions. The results show that logarithmic specifications produce a better fit, as reflected by higher R² and lower AIC/BIC values, yield realistic out-of-sample predictions (i.e., no negative emissions), and allow for elasticity interpretation without post-estimation. Second, the empirical contribution lies in providing the first comprehensive multi-strategy (USL, DSL, LBSL), multi-pollutant (CO, CO_₂_, HC, NO_x_, PMx, fuel), and multi-scenario (432 combinations) comparative evaluation under controlled experimental conditions. Strategy-specific quantification of impacts across pollutants reveals large differences in performance, such as a 46.8% increase in NO_x_ emissions due to LBSL relative to DSL. Third, the theoretical contribution establishes that emissions exhibit elastic responses to traffic volume (elasticities > 1) but inelastic responses to speed changes (elasticities < 1 in magnitude), with critical policy implications: demand management yields proportionally larger emission benefits than speed adjustments alone. The study also quantifies how heavy vehicle proportions moderate strategy effectiveness, demonstrating that fleet composition fundamentally shapes optimal policy choices. Together, these contributions address the identified gaps by providing transportation agencies with (a) a systematically compared analytical tools for policy evaluation, (b) empirical benchmarks for strategy comparison, and (c) practical guidance accounting for context-specific constraints.

### Study scope

This study analyzes data from 432 traffic simulation scenarios conducted on a 3.60 km highway segment, systematically varying speed limit strategy (DSL, LBSL, USL), posted speed limit (90, 100, 110, 120 km/h), traffic demand (500–1,750 veh/hr/lane), and heavy vehicle proportion (0–20%). Each scenario was simulated with 10 replications to ensure statistical reliability, yielding a robust dataset for regression analysis.

## Methodology

### Study framework

**Fig. 1:**
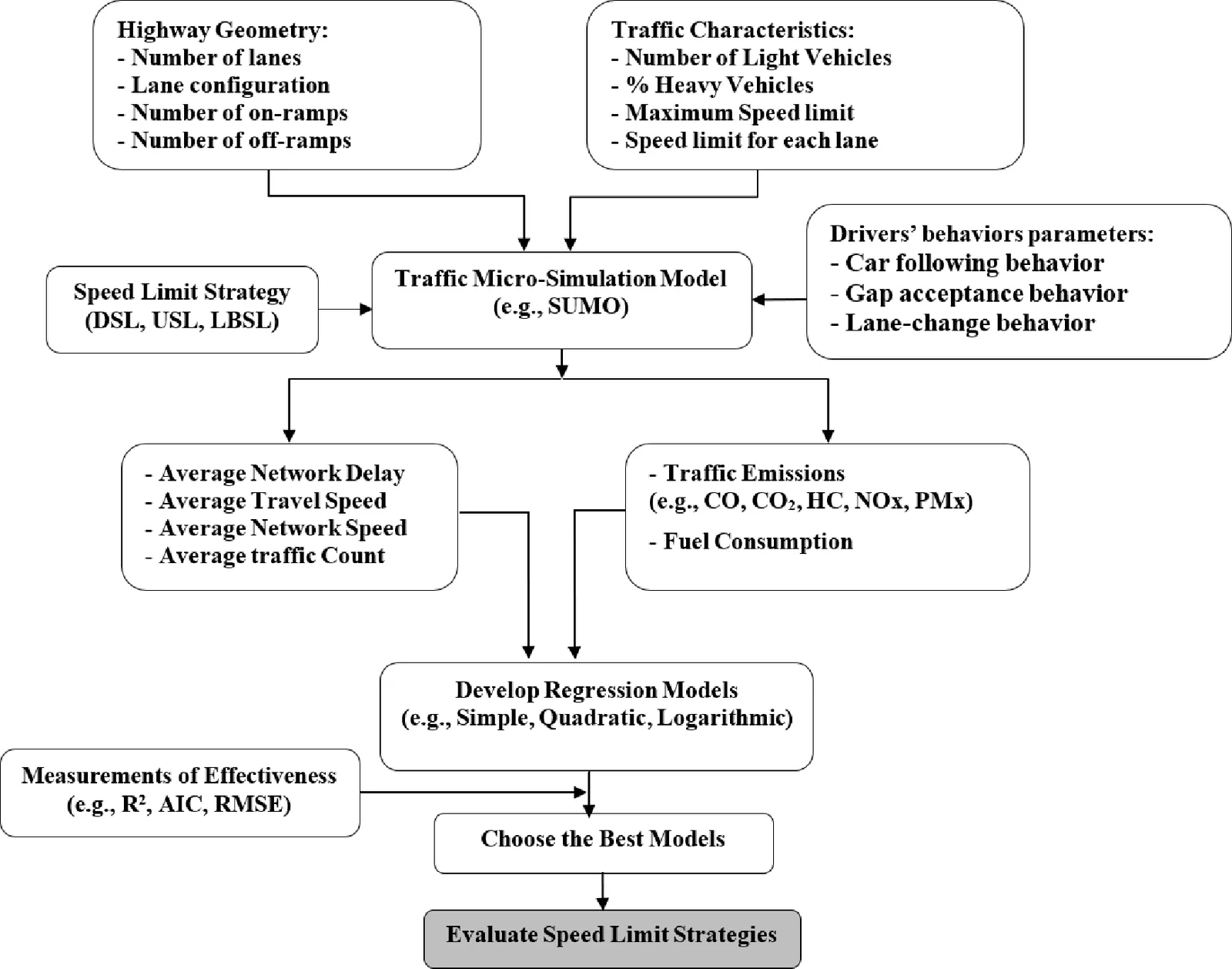
Study framework.

The analytical framework combines traffic microsimulation, emission modeling, and regression analysis to assess the performance of each strategy (see Fig. 1). The model takes as inputs the traffic demand, highway geometry, driver behavior parameters, vehicle fleet composition, and the speed limit strategy. These inputs are then passed to the SUMO microsimulation platform, which produces vehicle trajectories second-by-second. These trajectories are used to compute standard measures of network performance (average speed and delay) and environmental outcomes (emissions and fuel consumption) using established emission models. Finally, regression models estimate how inputs relate to outcomes, supporting consistent comparison of strategies.

### Network and simulation setup

**Fig. 2:**
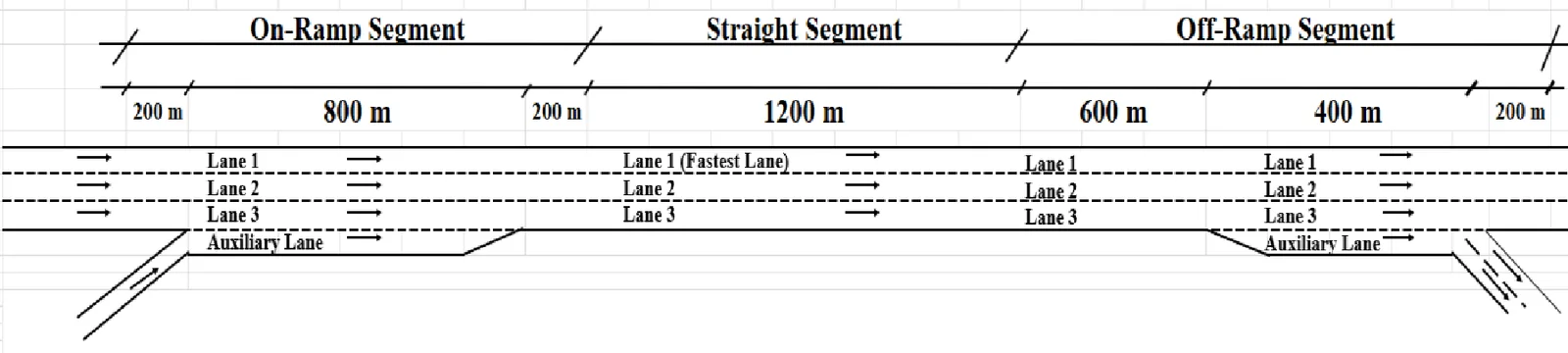
Layout of the study network.

A 3.6 km, three-lane highway segment was modeled, including a 1.2 km on-ramp weaving section, a 1.2 km middle section, and a 1.2 km off-ramp weaving section (see Fig. 2). Simulations were run in SUMO^35^. This study applied the Wiedemann-99 car-following model and the LC2013 lane-change model. The W99 parameters (CC1 through CC9) were adopted by Saccomanno et al^36^, who calibrated them against Next Generation Simulation (NGSIM) vehicle tracking data using genetic algorithms. The stand-still distance CC0 was adopted from Shahdah et al. (2022, 2026)^26,29^ recommended to reflect current driving behavior in Egypt (see Table 1). The emission factors were taken from the HBEFA database. HBEFA provides European-derived emission factors based on standard driving cycles and fleet composition assumptions^40^. While these factors are widely used in comparative simulation studies, they reflect European fleet characteristics and may not represent absolute emission levels in developing-country contexts with different fleet age distributions, fuel quality, and after-treatment technology. The traffic simulation was running for 90 min. Each scenario was run 10 times with different random seeds. The simulation ran for 15 min as a warm-up, and only the central 60 min of data were analyzed to avoid initialization and termination effects. Vehicle trajectory data, including position, speed, and acceleration profiles, were recorded.

**Table 1:** Simulation parameters.

Parameter	Adopted value	Units	Default value	Notes
Simulation step length	1.00	s	1.00	–
Light vehicle’s length	4.50	m	5.00	–
Heavy vehicle’s length	16.50	m	6.50	–
Car-following Model	W99	–	Krauss	–
CC0 (for light vehicles)	1.50	m	2.50	Stand-still gap
CC0 (for heavy vehicles)	2.50	m	2.50	Stand-still gap
CC1	1.09	s	1.30	Headway time
CC2	4.00	m	8.00	Following variation
CC3	−6.09	s	−12.00	Threshold for entering following regime
CC4	−0.35	m/s	−0.25	Negative following threshold
CC5	1.88	m/s	0.35	Positive following threshold
CC6	11.44	10^− 4^ rad/s	6.00	Speed dependency of oscillation
CC7	0.25	m/s^2^	0.25	Oscillation acceleration
CC8	3.50	m/s^2^	2.00	Standstill acceleration
CC9	1.50	m/s^2^	1.50	Desired acceleration at 80 km/h
Lane-Change Model	LC2013	–	LC2013	–

### Justifications for using microsimulation

Accurate emission estimation requires appropriate modeling methodologies that capture the complex relationships between traffic characteristics and emission rates. Emission models can be broadly categorized into three types: average-speed models, modal models, and instantaneous emission models^9^.

Average-speed models estimate emissions based on mean link or network speeds, applying emission factors that vary as functions of average speed. Examples include the COPERT model widely used in Europe^37^ and the MOBILE model series used in the United States^38^. These models are computationally efficient and suitable for large-scale planning applications but may not adequately capture the effects of speed variability, acceleration events, and transient operating conditions that significantly influence emissions^5^.

Modal models disaggregate emissions by vehicle operating mode, such as idle, acceleration, cruise, and deceleration. The MOVES (Motor Vehicle Emission Simulator) model developed by the U.S. EPA exemplifies this approach, providing more detailed representation of emission processes than average-speed models^39^. Modal models require more detailed input data, including the distribution of vehicle activity across operating modes, but offer improved accuracy for evaluating operational strategies.

Instantaneous emission models estimate emissions second by second from detailed vehicle trajectory data, so they can link emission rates to specific driving behaviors such as acceleration, braking, and speed changes. VT-Micro^18^, and PHEM^40^ fall into this category. Although these models can give the most accurate results, they depend on detailed vehicle trajectory data that usually comes from traffic microsimulation, which limits their use to specific corridors or selected networks.

Traffic microsimulation is often used to produce the detailed vehicle trajectory data that instantaneous emission models need. Simulation tools such as SUMO (Simulation of Urban Mobility) can represent vehicle behavior at a good resolution, which allows the model to capture changes in acceleration, braking, and speed variation patterns^35^. Combined with emission models, microsimulation allows researchers to assess how traffic management strategies, signal timing plans, and speed limit policies affect emissions^41^.

Combining traffic microsimulation with emission models can support the assessment of speed limit policies and other traffic management measures by linking detailed vehicle movements to estimated emissions. By simulating the trajectories of individual vehicles across different policy scenarios, researchers can track detailed speed and acceleration patterns that shape how emissions change, and this can produce estimates that are more accurate than those based on aggregate models^42^.

SUMO is an open-source traffic simulation tool that models individual vehicles and supports multiple travel modes. It is widely used in transportation research and applied studies^35^. SUMO uses physics-based models for car-following and lane-changing to produce realistic vehicle trajectories. These trajectories can then support emission estimates, using either SUMO’s built-in emission models or external ones. The platform integrates emission factors from Handbook Emission Factors for Road Transport (HBEFA) and supports user-defined emission calculations based on vehicle kinematics^43^.

Microsimulation is well-suited for comparing speed limit strategies because it can model how USL, DSL, and LBSL differ in lane use, speed distribution, and interactions between vehicles. Previous microsimulation studies report that traffic management options can lead to clear differences in emissions that are not captured by aggregate analyses^44,45^. Microsimulation studies on how speed limit strategies affect emissions are still limited, especially studies that compare several strategies under different traffic conditions and vehicle fleet compositions.

### Experimental design

A full-factorial experimental design was employed, resulting in 432 unique scenarios (Table 2). Scenarios systematically varied speed limit strategy (DSL (baseline), LBSL (successive lane reduction of 10 km/h), USL). Figure 4 illustrates the three common speed strategies. The posted speed limits vary from 90 to 120 km/h in 4 levels, traffic demand varies from 500 to 1,750 veh/h/lane in 6 levels, and heavy vehicle proportion varies from 0 to 20% in 6 levels. Each scenario was replicated 10 times with different random seeds for statistical reliability.

**Table 2:** Experimental design parameters and values.

Parameter	Values	Description
Strategies	DSL, LBSL, USL	Differential, Lane-Based, and Uniform Speed Limits
Speed Limits	90, 100, 110, 120 km/h	Posted speed limits
Demand Levels	500, 750, 1000, 1250, 1500, 1750 veh/hr/lane	Traffic demand
Trailer Percentages	0, 2.5, 5, 10, 15, 20%	Heavy vehicle proportion
Total Scenarios	432	3 × 4 × 6 × 6 combinations

**Fig. 3:**
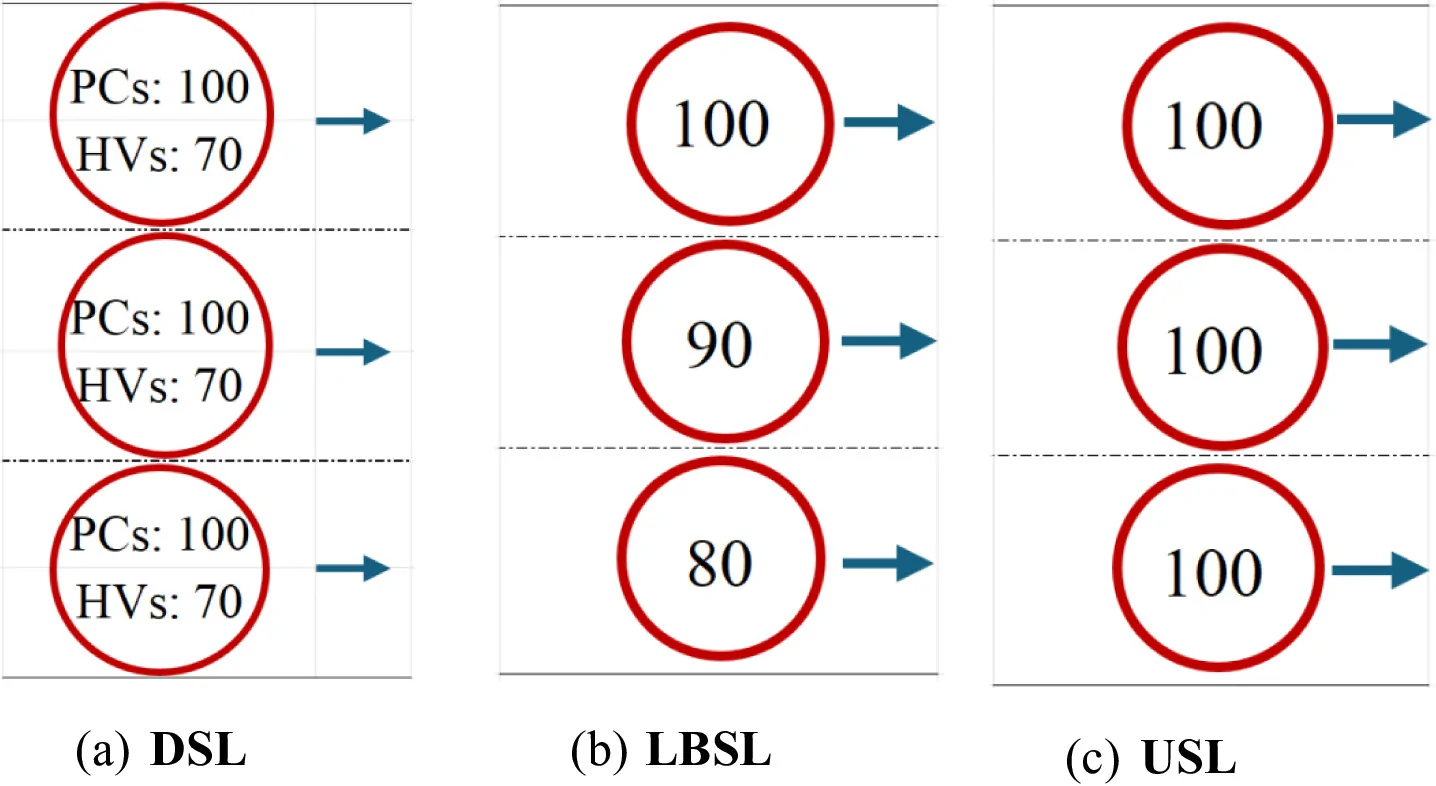
An example of DSL compared to LBSL and USL (Speed in km/h).

### Model specification

Post-simulation, average network speed, traffic count and total emissions/fuel consumption per kilometer were calculated. For each pollutant/fuel (dependent variable, Table 3), three regression forms were estimated against independent variables (Eqs. 1–3):

**Table 3:** Dependent and independent variables.

Variable	Symbol	Unit	Description
Dependent Variables			
CO emissions	* CO*	g/km	Carbon monoxide emissions per kilometer
CO₂ emissions	$\:C{O}_{2}$	g/km	Carbon dioxide emissions per kilometer
HC emissions	* HC*	g/km	Hydrocarbon emissions per kilometer
NO_x_ emissions	$\:N{O}_{x}$	g/km	Nitrogen oxide emissions per kilometer
PMx emissions	* PMx*	g/km	Particulate matter emissions per kilometer
Fuel consumption	* Fuel*	L/km	Fuel consumption per kilometer
Independent Variables			
Traffic count	* Q*	veh/hr	Traffic volume
Average speed	* S*	km/h	Mean traffic speed
Speed squared	$\:{S}^{2}$	(km/h)²	Quadratic speed term
Log traffic	$\:ln\left(V\right)$	log(veh/hr)	Natural logarithm of traffic count
Log speed	$\:ln\left(S\right)$	log(km/h)	Natural logarithm of speed
Trailer percentage	* T*	%	Proportion of heavy vehicles
Strategy LBSL	$\:{D}_{LBSL}$	0/1	Dummy variable for Lane-Based Speed Limit, 1 = LBSL strategy is implemented, 0 = otherwise
Strategy USL	$\:{D}_{USL}$	0/1	Dummy variable for Uniform Speed Limit, 1 = USL strategy is implemented, 0 = otherwise

From 1: Linear

1 $$\:Y\mathrm{=}{\beta\:}_{0}\mathrm{+}{\beta\:}_{1}V\mathrm{+}{\beta\:}_{2}S\mathrm{+}{\beta\:}_{3}T\mathrm{+}{\beta\:}_{4}{D}_{LBSL}\mathrm{+}{\beta\:}_{5}{D}_{USL}\mathrm{+}\epsilon\:$$

From 2: Quadratic

2 $$\:Y\mathrm{=}{\beta\:}_{0}\mathrm{+}{\beta\:}_{1}V\mathrm{+}{\beta\:}_{2}S\mathrm{+}{\beta\:}_{3}{S}^{2}\mathrm{+}{\beta\:}_{4}T\mathrm{+}{\beta\:}_{5}{D}_{LBSL}\mathrm{+}{\beta\:}_{6}{D}_{USL}\mathrm{+}\epsilon\:$$

Form 3: Logarithmic

3 $$\:ln\left(Y\right)\mathrm{=}{\beta\:}_{0}\mathrm{+}{\beta\:}_{1}ln\left(V\right)\mathrm{+}{\beta\:}_{2}ln\left(S\right)\mathrm{+}{\beta\:}_{3}T\mathrm{+}{\beta\:}_{4}{D}_{LBSL}\mathrm{+}{\beta\:}_{5}{D}_{USL}\mathrm{+}\epsilon\:$$

The DSL serves as the reference category for strategy comparisons.

Where:

* Y* = Emissions (g/km) or Fuel consumption (L/km),

* V* = Traffic volume (veh/hr),

* S* = Average speed (km/h),

$\:{S}^{2}$ = Speed squared term to capture non-linear speed effects,

* T* = Trailer percentage (%),

$\:{D}_{LBSL}$ = Dummy variable equals 1 if LBSL strategy, 0 otherwise,

$\:{D}_{USL}$ = Dummy variable equals 1 if USL strategy, 0 otherwise,

$$\:ln\left(\text{}\right)$$ = Natural logarithm,

$$\:\epsilon\:$$ = Error term.

All reported emissions and fuel values are normalized to grams (or liters, for fuel) per kilometer of road, computed as the total scenario-level emissions divided by the corridor length (3.6 km). The per-kilometer metric isolates the emission intensity of the traffic flow under different speed limit strategies; total corridor emissions scale linearly with the corridor length and therefore with the demand and the corridor extent.

### Software and statistical analysis

The statistical analyses and data visualizations in this study were completed in Python 3.11^46^. Data manipulation and preprocessing were carried out in pandas^47^. Numerical calculations, such as logarithmic transformations and array operations, were carried out in NumPy^48^. The regression analysis was run with the stats model’s library^49^. This library was used to fit all 18 regression models and to calculate coefficient estimates, standard errors, t-statistics, p-values, confidence intervals, and model diagnostics such as R², adjusted R², AIC, BIC, and F-statistics. Residual plots, Q–Q plots to check normality, scale-location plots to assess constant variance, and leverage plots to identify influential observations were produced with the package’s built-in diagnostic functions and SciPy^50^. Data visualizations were created in Python using Matplotlib^51^, and Seaborn^52^.

## Results and discussion

### Descriptive statistics of simulation outcomes

Before presenting the regression model results, we first examine the raw simulation outcomes to characterize the operational performance of the three speed limit strategies across the 432 scenarios. Tables 4, 5 and 6 summarize the mean, standard deviation, minimum, and maximum values for average network speed, average delay, and the six emission and fuel consumption metrics, disaggregated by strategy (DSL, LBSL, USL). All values are based on 144 simulation scenarios per strategy (total 432 scenarios). Speed and delay values are network-averaged over the 3.6 km corridor; emission and fuel values represent total corridor emissions per kilometer (g/km) aggregated across all vehicle types.

**Table 4:** Summary by strategy (*n* = 144 per strategy).

Measure	DSL (*n* = 144)	LBSL (*n* = 144)	USL (*n* = 144)
Average speed (km/h)	94.74 ± 16.56 (44.35–123.91)	83.04 ± 19.81 (39.60–115.24)	100.88 ± 13.18 (62.39–123.77)
CO (g/km)	13.564 ± 8.461 (2.819–32.536)	15.792 ± 9.345 (3.384–36.894)	12.605 ± 7.627 (2.752–30.404)
CO₂ (g/km)	1155.0 ± 941.1 (228.6–4770.1)	1660.1 ± 1322.8 (293.5–5633.7)	1092.7 ± 792.4 (228.5–4359.9)
HC (g/km)	0.1461 ± 0.1118 (0.0216–0.5673)	0.1879 ± 0.1458 (0.0272–0.6618)	0.1389 ± 0.1011 (0.0216–0.5350)
NOₓ (g/km)	2.736 ± 3.814 (0.090–21.094)	4.669 ± 5.753 (0.118–26.874)	2.918 ± 3.537 (0.090–20.231)
PMx (g/km)	0.1638 ± 0.0888 (0.0462–0.4764)	0.1793 ± 0.1117 (0.0430–0.5429)	0.1714 ± 0.0915 (0.0463–0.4660)
Fuel (L/km)	0.5034 ± 0.4095 (0.0999–2.0735)	0.7231 ± 0.5754 (0.1282–2.4478)	0.4760 ± 0.3446 (0.0998–1.8947)

* Mean ± Standard deviation (Minimum – Maximum).

**Table 5:** Traffic performance measures.

Strategy	Speed limit (km/h)	Avg speed (km/h)	Avg travel time (s)	Avg delay (s/veh)
DSL	90	79.46 ± 12.18	167.65 ± 34.73	25.44 ± 32.87
	100	89.91 ± 12.29	147.07 ± 27.71	19.88 ± 26.31
	110	99.96 ± 12.34	131.45 ± 21.69	16.40 ± 20.64
	120	109.65 ± 12.17	119.19 ± 17.00	14.07 ± 16.15
LBSL	90	69.77 ± 14.54	195.43 ± 56.35	49.79 ± 53.03
	100	78.15 ± 16.43	174.64 ± 50.87	44.59 ± 48.41
	110	87.38 ± 18.09	155.88 ± 44.97	38.38 ± 43.10
	120	96.88 ± 19.29	139.90 ± 38.64	32.59 ± 37.19
USL	90	86.21 ± 7.32	150.09 ± 14.95	13.83 ± 14.96
	100	95.91 ± 7.74	134.77 ± 12.48	12.17 ± 12.49
	110	105.85 ± 7.22	121.82 ± 8.90	10.37 ± 8.91
	120	115.57 ± 7.11	111.46 ± 7.16	9.23 ± 7.16

* Mean ± Standard deviation.

**Table 6:** Emission intensity (g/km and L/km).

Strategy	Speed limit (km/h)	CO	CO₂	HC	NOₓ	PMx	Fuel
DSL	90	11.523 ± 7.774	1165.0 ± 1009.2	0.1370 ± 0.1132	2.809 ± 3.894	0.1576 ± 0.0890	0.5077 ± 0.4392
	100	12.690 ± 8.088	1150.1 ± 993.2	0.1423 ± 0.1158	2.817 ± 4.123	0.1609 ± 0.0903	0.5012 ± 0.4321
	110	14.192 ± 8.579	1147.1 ± 928.2	0.1487 ± 0.1124	2.708 ± 3.828	0.1657 ± 0.0894	0.4999 ± 0.4038
	120	15.851 ± 9.066	1158.1 ± 866.9	0.1565 ± 0.1096	2.612 ± 3.550	0.1711 ± 0.0896	0.5048 ± 0.3772
LBSL	90	13.680 ± 8.331	1712.0 ± 1400.6	0.1804 ± 0.1502	4.968 ± 6.191	0.1735 ± 0.1155	0.7456 ± 0.6092
	100	15.173 ± 9.060	1686.2 ± 1368.2	0.1863 ± 0.1490	4.792 ± 5.964	0.1771 ± 0.1137	0.7344 ± 0.5951
	110	16.457 ± 9.587	1640.5 ± 1314.3	0.1903 ± 0.1461	4.576 ± 5.680	0.1810 ± 0.1116	0.7146 ± 0.5717
	120	17.856 ± 10.178	1601.5 ± 1257.4	0.1946 ± 0.1435	4.341 ± 5.370	0.1855 ± 0.1102	0.6977 ± 0.5470
USL	90	10.347 ± 6.764	1088.7 ± 898.6	0.1292 ± 0.1095	3.126 ± 4.121	0.1628 ± 0.0922	0.4742 ± 0.3907
	100	11.819 ± 7.306	1089.8 ± 842.2	0.1355 ± 0.1055	2.995 ± 3.797	0.1678 ± 0.0918	0.4747 ± 0.3662
	110	13.296 ± 7.676	1087.0 ± 747.4	0.1414 ± 0.0976	2.832 ± 3.294	0.1738 ± 0.0915	0.4736 ± 0.3251
	120	14.957 ± 8.218	1105.2 ± 699.9	0.1495 ± 0.0943	2.718 ± 2.958	0.1811 ± 0.0934	0.4817 ± 0.3045

* Mean ± Standard deviation.

As shown in Table 4, the three strategies generated distinct speed profiles across the 144 scenarios per strategy. USL produces the highest average speed with LBSL at the opposite extreme and DSL intermediate, as confirmed by the mean speeds of 100.88, 94.74, and 83.04 km/h for USL, DSL, and LBSL respectively. Travel time and delay comparisons across strategies are presented in Table 5 and discussed in the following paragraph. The substantially larger standard deviation of delay under LBSL (averaged across all four speed limit settings: ±45.4 s vs. ± 10.9 s for USL) is consistent with the inter-lane speed differentiation creating heterogeneous operating conditions.

As shown in Table 5, for all three strategies, raising the speed limit from 90 to 120 km/h produces higher average speeds, shorter travel times, and lower delays. The speed gain is largest for DSL (+ 30.2 km/h from 90 to 120) and smallest for LBSL (+ 27.1 km/h), with USL intermediate (+ 29.4 km/h), but the gap to the speed limit is largest for LBSL (its mean speed at the 90 km/h setting is only 69.8 km/h, while USL at 90 is 86.2 km/h, indicating that LBSL vehicles run furthest below the posted limit on average). Delay under LBSL is 3–4× higher than under USL at every speed limit, with a stronger dependence on the speed limit setting (LBSL delay drops from 49.8 s at 90 km/h to 32.6 s at 120 km/h, a 35% reduction; USL delay drops from 13.8 s to 9.2 s, a 33% reduction; DSL delay drops from 25.4 s to 14.1 s, a 45% reduction).

As shown in Table 6:

- *CO* increases with speed limit across all three strategies (11.52 → 15.85 g/km for DSL, 13.68 → 17.86 g/km for LBSL, 10.35 → 14.96 g/km for USL). LBSL is highest at every speed limit; USL is lowest; DSL is intermediate.
- *CO₂* is approximately constant for DSL (1147–1165 g/km across speed limits), decreases slightly with speed for LBSL (1712 → 1602 g/km from 90 to 120), and is approximately constant for USL (1087–1106 g/km). LBSL is consistently 45–57% above USL at every speed limit.
- *HC* shows the same pattern as CO, increasing with speed limit and with strategy ranking USL < DSL < LBSL.
- *NO*$_{x}$ decreases with speed limit for all three strategies (DSL: 2.81 → 2.61; LBSL: 4.97 → 4.34; USL: 3.13 → 2.72 g/km), consistent with reduced stop-and-go behavior, lower acceleration intensity, and smoother traffic flow at higher speed limit settings, which reduce the transient engine events that drive NO_x_ formation. LBSL is approximately 59–62% above USL at every speed limit (58.9% at 90 km/h, 60.0% at 100 km/h, 61.6% at 110 km/h, and 59.7% at 120 km/h).
- *PMx* increases with speed for all three strategies; the LBSL–USL gap is small (2–7% across speed limits).
- *Fuel* is approximately constant for DSL (0.500–0.508 L/km), decreases with speed for LBSL (0.746 → 0.698 L/km from 90 to 120), and is approximately constant for USL (0.474–0.482 L/km, with values ranging from 0.4736 at 110 km/h to 0.4817 at 120 km/h).

For CO and HC, the absolute gap between strategies does not narrow monotonically with increasing speed limit: the LBSL–DSL gap widens slightly from the 90 to 100 km/h setting before narrowing at higher speed limits. The narrowing is consistent only from the 100 km/h setting onward for these two pollutants. For PMx, raising the speed limit consistently narrows the gap between strategies across all speed limit settings. For CO₂ and fuel, the USL–DSL gap narrows with increasing speed limit, while LBSL remains consistently elevated. This interaction effect is consistent with the log-log specification of the regression models and is captured by the model (refer to Sect. "Regression model performance").

### Regression model performance

#### Bivariate exploratory analysis

Before estimating the full multivariate specifications, bivariate scatter plots were generated for each dependent variable against the two primary continuous predictors, average traffic count and average network speed, under all three functional forms (linear, quadratic, and log-log). The intention was to obtain a preliminary, visual and quantitative assessment of which functional form best captures the univariate relationships between emissions (or fuel consumption) per kilometer and each predictor individually.

Table 7 reports the coefficients of determination (R²) from these bivariate regressions. Two clear and instructive patterns emerge from the table. Pattern 1, traffic count as the predictor: For every pollutant and for fuel consumption, the log-log formulation (ln(pollutant) regressed on ln(traffic count)) yields the highest bivariate R². The log-log advantage is substantial: for CO, the log-log R² reaches 0.913 compared with 0.871 for the linear form; for CO₂, the gain is from 0.617 (linear) to 0.829 (log-log); for NOₓ, it rises from 0.271 to 0.324; and for fuel consumption, from 0.618 to 0.830. This outcome aligns with first-principles reasoning: emissions and fuel consumption are, to a first approximation, proportional to traffic volume, doubling the number of vehicles approximately doubles the total emissions produced per unit length of roadway. A proportional relationship of this kind is inherently multiplicative, and the logarithmic transformation linearizes it. The near-unity traffic-volume elasticity that the log-log model will later recover (see Table 7) is foreshadowed by these bivariate results.

**Table 7:** Coefficients of determination (R²) from bivariate regressions of emissions (g/km) and fuel consumption against traffic count (veh/hr) and average network speed.

Variable	X-axis	Linear	Quadratic	Log-Log
CO (g/km)	Traffic Count (veh/hr)	0.871	0.909	**0.913**
	Speed (km/h)	**0.156**	0.123	0.139
CO₂ (g/km)	Traffic Count (veh/hr)	0.617	0.659	**0.829**
	Speed (km/h)	**0.501**	0.422	0.500
HC (g/km)	Traffic Count (veh/hr)	0.589	0.619	**0.767**
	Speed (km/h)	**0.293**	0.244	0.249
NOₓ (g/km)	Traffic Count (veh/hr)	0.271	0.291	**0.324**
	Speed (km/h)	**0.260**	0.220	0.146
PMx (g/km)	Traffic Count (veh/hr)	0.537	0.537	**0.664**
	Speed (km/h)	**0.037**	0.024	0.029
Fuel (L/km)	Traffic Count (veh/hr)	0.618	0.661	**0.830**
	Speed (km/h)	**0.502**	0.423	0.501

Bold values indicate the highest R² achieved among all methods for each variable (row)

Pattern 2, speed as the predictor: When the same dependent variables are regressed against average network speed alone, the linear form produces marginally higher R² values than the logarithmic or quadratic forms for most pollutants (e.g., CO₂: linear R² = 0.501 vs. log-log R² = 0.500; NOₓ: linear R² = 0.260 vs. log-log R² = 0.146; HC: linear R² = 0.293 vs. log-log R² = 0.249). However, the absolute magnitudes of these speed-only R² values are markedly lower than those obtained with traffic count across all three functional forms and all six dependent variables. For example, the speed-only R² for NOₓ never exceeds 0.260, whereas the traffic-count R² for the same pollutant reaches 0.324 under the log-log form. Similarly, for PMx, speed explains at most 3.7% of the variance (linear R² = 0.037) while traffic count explains up to 66.4% (log-log R² = 0.664). This asymmetry, traffic count dominating speed as a predictor of per-kilometer emission intensity, is consistent with the physical understanding that emission totals are driven primarily by the quantity of traffic, with speed playing a modulating, second-order role. The fact that the linear form marginally outperforms the log-log form for speed-only regressions suggests that the speed–emission relationship, in isolation, is not strictly multiplicative; rather, it reflects the combined influence of several offsetting mechanisms (engine load, gear selection, cruising efficiency) whose net effect in the narrow operating range of 90–120 km/h is approximately linear.

The bivariate evidence demonstrates that no single functional form dominates both predictors simultaneously, the log-log form is superior for traffic count, while the linear form has a modest edge for speed. This finding validates the decision to estimate full multivariate specifications in which both predictors enter the model simultaneously and compete to explain the variance, with the overall model fit determining which functional form is ultimately preferred.

#### Full multivariate model result

Table 8 presents the goodness-of-fit metrics for the 18 estimated regression models (six dependent variables × three functional forms: linear, quadratic, and logarithmic). The multivariate results resolve the ambiguity present in the bivariate analysis: the logarithmic specification consistently and substantially outperforms both the linear and quadratic forms across all six dependent variables on the scale-invariant criteria (R², adjusted R², and F-statistic). In addition, Table 9 reports the estimated coefficients, standard errors, and significance levels for all 18 models. Only one coefficient across the entire table, the intercept of the linear HC model, is not statistically significant at the 5% level; all other coefficients are significant at *p* < 0.05, and the vast majority are significant at *p* < 0.001. This near-universal significance reflects both the large sample size (*n* = 432 simulation scenarios) and the strong structural relationships embedded in the data.

**Table 8:** Goodness-of-fit metrics for linear, quadratic, and log regression models.

Y	Model type	*R*²	Adj. *R*²	AIC	BIC	RMSE	F-statistic	*p*-value
CO	Linear	0.90	0.90	2090.39	2114.80	2.70	786.99	< 0.001
CO	Quadratic	0.92	0.92	1995.55	2024.03	2.42	836.49	< 0.001
CO	Logarithmic	0.94	0.94	−337.35	−312.94	0.16	1388.86	< 0.001
CO_2_	Linear	0.82	0.82	6526.31	6546.65	458.93	480.25	< 0.001
CO_2_	Quadratic	0.91	0.91	6234.01	6258.42	326.84	840.71	< 0.001
CO_2_	Logarithmic	0.97	0.97	−479.58	−459.24	0.14	3092.74	< 0.001
HC	Linear	0.81	0.81	−1290.61	−1270.27	0.05	449.37	< 0.001
HC	Quadratic	0.86	0.86	−1435.98	−1411.57	0.05	539.10	< 0.001
HC	Logarithmic	0.95	0.95	−369.19	−348.85	0.16	2261.34	< 0.001
NO_x_	Linear	0.68	0.68	2050.74	2075.15	2.58	183.15	< 0.001
NO_x_	Quadratic	0.75	0.75	1946.98	1975.46	2.29	214.15	< 0.001
NO_x_	Logarithmic	0.91	0.91	379.29	403.70	0.37	863.96	< 0.001
PMx	Linear	0.86	0.86	−1631.20	−1606.79	0.04	538.09	< 0.001
PMx	Quadratic	0.89	0.89	−1727.53	−1699.05	0.03	579.80	< 0.001
PMx	Logarithmic	0.97	0.97	−875.48	−851.07	0.09	3313.69	< 0.001
Fuel	Linear	0.82	0.82	5805.70	5826.04	199.31	482.23	< 0.001
Fuel	Quadratic	0.91	0.91	5511.96	5536.38	141.71	846.93	< 0.001
Fuel	Logarithmic	0.97	0.97	−480.01	−459.67	0.14	3090.93	< 0.001

**Table 9:** Models’ coefficients.

Variable	CO			CO_2_			HC		
	Simple	Quadratic	Log	Simple	Quadratic	Log	Simple	Quadratic	Log
Intercept	−10.96 [1.32]^**a**^	8.6229 [2.23]^**a**^	−11.7607 [0.35]^**a**^	902.8418 [223.83]^**a**^	6132.8049 [301.95]^**a**^	−2.8645 [0.29]^**a**^	−0.0091 [0.03]^**b**^	0.4618 [0.04]^**a**^	−13.7596 [0.33]^**a**^
Strategy LBSL	2.6926 [0.34]^**a**^	2.5177 [0.30]^**a**^	0.2136 [0.02]^**a**^	265.9001 [53.04]^**a**^	230.4397 [37.81]^**a**^	0.2407 [0.02]^**a**^	0.0278 [0.01]^**a**^	0.0246 [0.01]^**a**^	0.2171 [0.02]^**a**^
Strategy USL	−1.1824 [0.32]^**a**^	−1.2841 [0.29]^**a**^	−0.0795 [0.02]^**a**^	–	^–^	^–^	^–^	^–^	^–^
Traffic Volume	0.0066 [0.00]^a^	0.0066 [0.00]^a^	1.6005 [0.02]^a^	0.5132 [0.02]^**a**^	0.5030 [0.02]^**a**^	1.4765 [0.02]^**a**^	0.0001 [0.00]^**a**^	0.0001 [0.00]^**a**^	1.4844 [0.02]^**a**^
Speed	0.0300 [0.01]^**a**^	−0.4400 [0.05]^**a**^	0.2919 [0.05]^**a**^	−19.0017 [1.67]^**a**^	−144.4975 [6.27]^**a**^	−0.5417 [0.04]^**a**^	−0.0013 [0.00]^**a**^	−0.0126 [0.00]a	−0.1600 [0.04]^**a**^
Speed ^2^	–	0.0027 [0.00]^**a**^	–	–	0.7234 [0.04]^**a**^	–	–	0.0001 [0.00]^**a**^	–
Trailer %	−0.0557 [0.02]^**a**^	−0.0596 [0.02]^**a**^	−0.0042 [0.00]^**a**^	40.0204 [3.17]^**a**^	38.9416 [2.26]^**a**^	0.0278 [0.00]^**a**^	0.0068 [0.00]^**a**^	0.0067 [0.00]^**a**^	0.0415 [0.00]^**a**^

Variable	No _x_			PM _x_			Fuel		
	Simple	Quadratic	Log	Simple	Quadratic	Log	Simple	Quadratic	Log
Intercept	2.8961 [1.26]^**a**^	22.3507 [2.11]^**a**^	−9.4798 [0.79]^a^	−0.0417 [0.02]^a^	0.2239 [0.03]^**a**^	−10.4242 [0.19]^**a**^	393.0947 [97.21]^**a**^	2668.2920 [130.92]^**a**^	−3.6943 [0.29]^**a**^
Strategy LBSL	1.0384 [0.32]^a^	0.8646 [0.29]^**a**^	0.3839 [0.05]^a^	0.0112 [0.00]^**a**^	0.0088 [0.00]^**a**^	0.0442 [0.01]^**a**^	115.7938 [23.03]^**a**^	100.3674 [16.39]^**a**^	0.2404 [0.02]^**a**^
Strategy USL	0.6545 [0.31]^**a**^	0.5534 [0.27]^**a**^	0.1375 [0.04]^a^	0.0099 [0.00]^**a**^	0.0086 [0.00]^**a**^	0.0451 [0.01]^**a**^	^–^	^–^	^–^
Traffic Volume	0.0013 [0.00]^a^	0.0012 [0.00]^a^	1.4833 [0.05]^a^	0.0001 [0.00]^a^	0.0001 [0.00]^a^	1.0422 [0.01]^a^	0.2236 [0.01]^a^	0.2192 [0.01]^**a**^	1.4764 [0.02]^**a**^
Speed	−0.0783 [0.01]^**a**^	−0.5451 [0.04]^**a**^	−0.7089 [0.11]^**a**^	−0.0004 [0.00]^**a**^	−0.0068 [0.00]^**a**^	−0.0603 [0.02]^**a**^	−8.2644 [0.72]^**a**^	−62.8590 [2.72]^**a**^	−0.5411 [0.04]^**a**^
Speed ^2^	–	0.0027 [0.00]^**a**^	–	–	0.0000 [0.00]^**a**^	–	–	0.3147 [0.02]^**a**^	–
Trailer %	0.3466 [0.02]^**a**^	0.3427 [0.02]^**a**^	0.1276 [0.00]^**a**^	0.0077 [0.00]^**a**^	0.0077 [0.00]^**a**^	0.0431 [0.00]^**a**^	17.3033 [1.38]^**a**^	16.8340 [0.98]^**a**^	0.0276 [0.00]^**a**^

Values between [] represent the standard error, ^a^: the coefficient is significant at 5% level, and ^b^: the coefficient is not significant at 5% level.

The log-log model achieves the highest R² and adjusted R² for every pollutant and for fuel consumption. The R² values for the logarithmic models range from 0.91 (NOₓ) to 0.97 (CO₂, PMx, Fuel), indicating that the model explains 91–97% of the variance in the log-transformed dependent variables. By comparison, the linear models achieve R² values ranging from 0.68 (NOₓ) to 0.90 (CO), while the quadratic models occupy an intermediate position (R² = 0.75 to 0.92). The improvement of the logarithmic form over the linear form is most dramatic for NOₓ, where R² rises from 0.68 to 0.91, a 23-percentage-point gain, and for CO₂ and Fuel, where R² increases by 15% points (0.82 to 0.97). The adjusted R² values, which penalize the inclusion of additional parameters, mirror the R² rankings exactly, confirming that the superiority of the logarithmic model is not an artifact of overfitting.

The AIC (Akaike Information Criterion) and BIC (Bayesian Information Criterion) trade off goodness-of-fit against model complexity, with lower values preferred. These criteria can be compared across functional forms only when the dependent variable is on the same scale; because the logarithmic models are fitted to ln(y) rather than y, their raw AIC/BIC values are not directly comparable to those of the linear and quadratic models (this point is elaborated in Sect. "Methodological notes on model comparison"). Where the dependent variable is untransformed, the AIC/BIC clearly favor the more flexible specifications: for CO₂ the quadratic AIC (6234.01) is 292 units below the linear (6526.31), and for Fuel the quadratic AIC (5511.96) is 294 units below the linear (5805.70). For HC and PMx, the quadratic specification attains the lowest raw AIC/BIC of all three forms (HC: −1435.98 vs. −1290.61 linear and − 369.19 log; PMx: −1727.53 vs. −1631.20 linear and − 875.48 log), but, as noted above, the log value is on a different scale and cannot be ranked against the others. Consequently, model selection across functional forms is based on the scale-invariant criteria below rather than on raw AIC/BIC.

All 18 models are globally significant, with F-statistic and P-values below 0.001. The F-statistics for the logarithmic models are consistently the largest (e.g., CO₂: F = 3,093 for log-log vs. 841 for quadratic vs. 480 for linear), reflecting the greater explanatory power of the log-transformed specification after accounting for the degrees of freedom consumed. Combined with the uniformly highest R² and adjusted R², this makes the logarithmic form the preferred specification for every dependent variable.

The quadratic model, which adds a squared speed term (S²) to the linear specification, improves upon the linear model for every dependent variable, raising R² by 2 to 9% points and reducing AIC by approximately 95 to 295 units, but never surpasses the logarithmic form in R², adjusted R², or F. This pattern reveals that the quadratic term captures the curvature of the speed–emission relationship (the fact that emission rates are not strictly linear in speed) but does not address the fundamental structural characteristic of emission data: that emission generation is a multiplicative process. The log transformation addresses this structural property directly, making it the appropriate functional form on both theoretical and empirical grounds.

#### Methodological notes on model comparison

Two caveats must be borne in mind when interpreting the metrics in Table 8. First, the RMSE values for the logarithmic models are expressed in units of the log-transformed dependent variable, i.e., ln(g/km) for emissions and ln(L/km) for fuel, while the RMSE values for the linear and quadratic models are in the original units (g/km or L/km). The two are not directly comparable; a log-scale RMSE of 0.14 for CO₂, for instance, corresponds to an approximately 15% typical percentage error in the original scale, which is informative but cannot be numerically ranked alongside the linear-model RMSE of 459 g/km. When back-transformed, the log-model predictive errors in original units are considerably smaller than those of the linear and quadratic models, consistent with the R² and F-statistic rankings.

Second, the negative AIC and BIC values observed for several logarithmic models (and for the linear and quadratic HC and PMx models) are a consequence of the log-transformation of the dependent variable: the concentrated log-likelihood incorporates a Jacobian term that shifts the likelihood scale downward. These negative values carry no substantive meaning in themselves and do not affect the validity of relative model comparisons, which depend only on the differences in AIC/BIC between models fitted to the same dependent variable on the same scale. Because the logarithmic models are fitted to ln(y) while the linear and quadratic models are fitted to y, the AIC/BIC of the log models cannot be ranked against those of the untransformed models; the cross-form comparisons in Sect."Full Multivariate Model Result" therefore rely on the scale-invariant R², adjusted R², and F-statistic rather than on AIC/BIC.

In addition, the log model is more robust than the other two models in predicting dependent variables with independent variables outside the values used in the calibration of the models. For example, for Traffic volume = 1,200 veh/hr, Speed = 95 km/h, Trailer = 8%, and Strategy = DSL. The CO₂ Emissions (g/km) using the simple, quadratic, and log forms can be shown in Eqs. 4–6, respectively. The simple model yields an unrealistically low value (≈ 34 g/km), the quadratic model produces a negative CO₂ prediction (≈ − 151 g/km), while the log model yields a realistic positive value (≈ 213 g/km) (Eqs. 4–6). In addition, CO₂ typically ranges from about 137 g/km to about 274 g/km in observational studies of Euro-5/−6 and mixed fuel cars^53^^,^^54^; the log-model prediction falls squarely within this range. Consequently, all subsequent analyses are based on the log model results. Table 10 shows the coefficient interpretation for the log model.

4 $$\:\begin{array}{c}{CO}_{2}\mathrm{=}\text{}902.84177\mathrm{+}0.51321\times\:1200\mathrm{+}\left(\mathrm{-}19.00167\right)\times\:95\mathrm{+}40.02035\times\:8\\\:{CO}_{2}\text{}\mathrm{=}\text{}33.70g/km\end{array}$$

5 $$\:\begin{array}{c}{CO}_{2}\mathrm{=}\text{}6132.80\mathrm{+}0.503\times\:1200\mathrm{+}\left(\mathrm{-}144.497\right)\times\:95\mathrm{+}0.7234\times\:9025\mathrm{+}38.9416\times\:8\\\:{CO}_{2}\text{}\mathrm{=}\text{}\mathrm{-}150.58g/km\end{array}$$

6 $$\:\begin{array}{c}ln\left({CO}_{2}\right)\mathrm{=}\text{}\mathrm{-}2.86452\mathrm{+}1.47648\times\:7.0901\mathrm{+}\left(\mathrm{-}0.54165\right)\times\:4.5539\mathrm{+}0.02781\times\:8\mathrm{=}5.35957\\\:{CO}_{2}\mathrm{=}{e}^{5.35957}\text{}\mathrm{=}\text{}212.73g/km\end{array}$$

**Table 10:** Coefficient interpretation for the logarithmic model.

Coefficient	Interpretation	Formula
$\:{\beta\:}_{ln\left(V\right)}$	% change in Y per 1% change in traffic	$\:\mathrm{\%}\varDelta\:Y\mathrm{=}{\beta\:}_{i}\mathrm{.\%}\varDelta\:X$
$\:{\beta\:}_{ln\left(S\right)}$	% change in Y per 1% change in speed	$\:\mathrm{\%}\varDelta\:Y\mathrm{=}{\beta\:}_{i}\mathrm{.\%}\varDelta\:X$
$\:{\beta\:}_{T}$	% change in Y per 1 unit change in T	$\:\mathrm{\%}\varDelta\:Y\mathrm{=}\left({e}^{\beta\:}\mathrm{-}1\right)\times\:100$
$\:{\beta\:}_{LBSL}$	% difference from DSL	$\:\mathrm{\%}\varDelta\:Y\mathrm{=}\left({e}^{\beta\:}\mathrm{-}1\right)\times\:100$
$\:{\beta\:}_{USL}$	% difference from DSL	$\:\mathrm{\%}\varDelta\:Y\mathrm{=}\left({e}^{\beta\:}\mathrm{-}1\right)\times\:100$

#### Model diagnostic assessment

Beyond the aggregate goodness-of-fit metrics presented in Table 8, a thorough regression analysis requires verifying that the estimated models satisfy the underlying assumptions of ordinary least squares (OLS). Violations of different OLS assumptions carry distinct consequences: functional form misspecification (non-linearity) biases and renders inconsistent the coefficient estimates themselves, while violations of homoscedasticity (constant error variance), normality of the residuals, and the presence of unduly influential observations primarily invalidate the standard errors, confidence intervals, and hypothesis tests on which inference depends. Additionally, unduly influential observations can distort coefficient estimates by exerting disproportionate pull on the fitted surface. In transportation emission modeling, where the dependent variable is inherently the product of traffic volume, per-vehicle fuel rate, and emission factors, these assumptions are particularly consequential because forcing a multiplicative process into an additive model structure produces predictable and systematic violations.

**Fig. 4:**
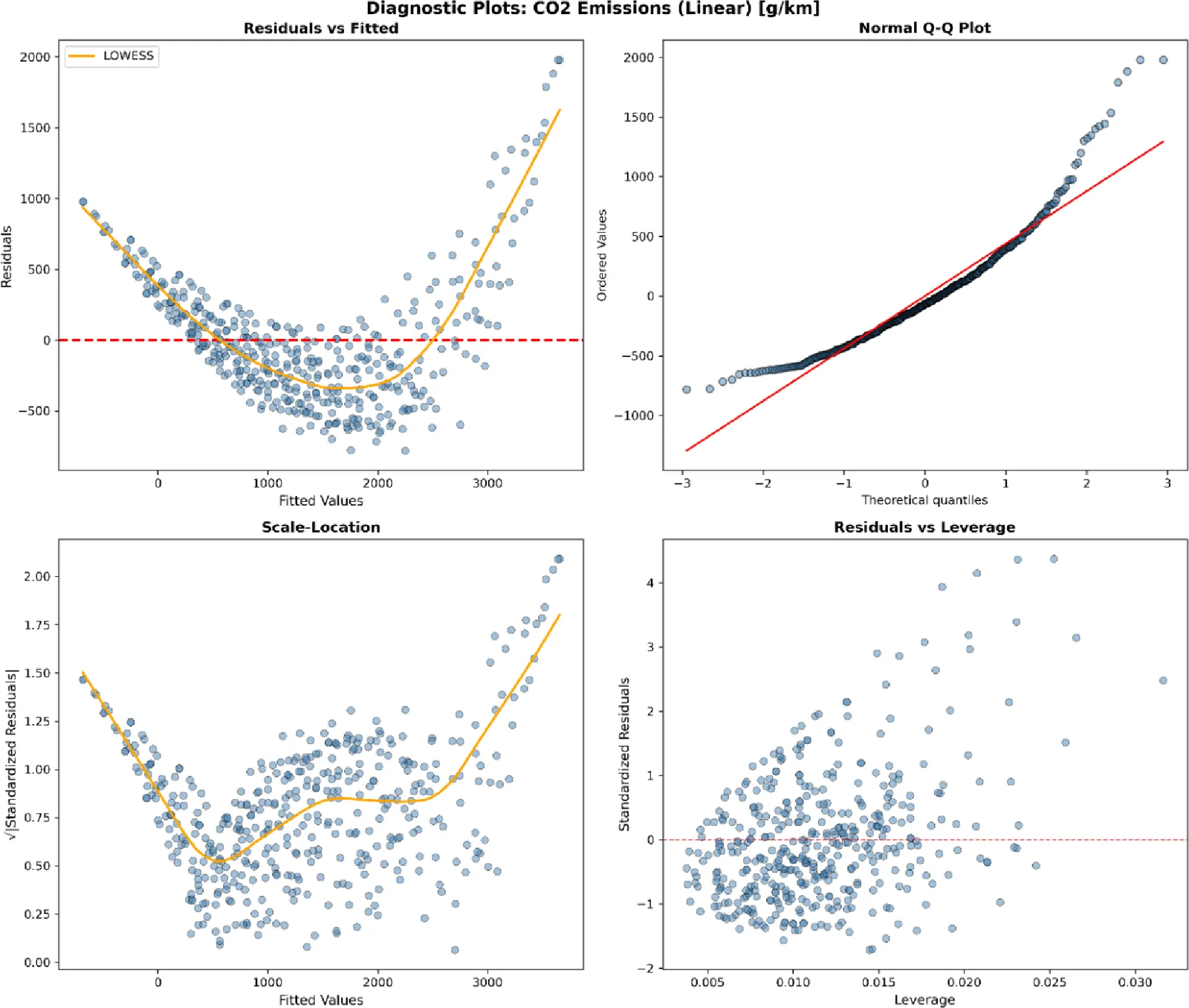
Diagnostic plots for the linear regression model fitted to CO₂ emissions per kilometer (g/km).

To assess assumption satisfaction, a standard four-panel diagnostic suite was generated for each of the three functional forms fitted to every dependent variable. The diagnostic panels for CO₂, presented in Figs. 4 (linear), 5 (quadratic), and 6 (logarithmic), comprise:

1. *Residuals vs. Fitted values* assess linearity and homoscedasticity. Residuals should scatter randomly around the horizontal zero line with no discernible pattern, curvature, or systematic change in vertical dispersion.
2. *Normal Q–Q plot of the standardized residuals* evaluates the normality assumption. Ordered standardized residuals lying along the 45° reference line indicate approximate normality; systematic curved (concave or convex) departures signal skewness, while S-shaped departures indicate that the residuals have fewer extreme values than a normal distribution would predict.
3. *Scale-Location plot* ($\:\surd\:\left|\boldsymbol{s}\boldsymbol{t}\boldsymbol{a}\boldsymbol{n}\boldsymbol{d}\boldsymbol{a}\boldsymbol{r}\boldsymbol{d}\boldsymbol{i}\boldsymbol{z}\boldsymbol{e}\boldsymbol{d}\:\boldsymbol{r}\boldsymbol{e}\boldsymbol{s}\boldsymbol{i}\boldsymbol{d}\boldsymbol{u}\boldsymbol{a}\boldsymbol{l}\boldsymbol{s}\right|\:$vs. fitted values): a complementary diagnostic for heteroscedasticity. The square-root transformation of the absolute standardized residuals dampens the visual dominance of extreme points. A horizontal Locally Weighted Scatterplot Smoothing (LOWESS) curve indicates constant error variance; a systematic upward or downward trend signals non-constant variance.
4. *Residuals vs. Leverage plot with Cook’s distance contours *identify observations that exert disproportionate influence on the fitted model. Points falling beyond the Cook’s distance threshold of 0.5 warrant scrutiny; those beyond 1.0 are considered potentially problematic.

CO₂ was selected as the representative pollutant for the diagnostic figures because it exhibits the widest range of emission values across the 432 simulation scenarios (228.5–5,633.7 g/km; Table 4), making the consequences of functional-form misspecification most visually apparent. The diagnostic suite was examined for all six dependent variables; the patterns described below are consistent across pollutants, with CO₂ displaying the most pronounced manifestations.

##### Linear model diagnostics (CO₂) (Fig. 4)

The linear specification, which regresses untransformed per-kilometer CO₂ emissions on untransformed traffic volume, speed, trailer percentage, and strategy dummies, exhibits clear violations of the homoscedasticity assumption.

*Residuals vs. Fitted:* The plot reveals a U-shaped variance pattern: the residual spread is widest at both low and high fitted emission values, while the central range displays a tighter dispersion. This pattern is a structural consequence of the mismatch between the linear model’s additive form and the multiplicative nature of the emission process. Per-kilometer CO₂ emissions are the product of traffic volume and per-vehicle fuel consumption. When this product is approximated by a linear additive function, the approximation error is smallest in the central range of the data, where the locally linear approximation to the multiplicative surface is most accurate, and largest at both extremes, where the curvature of the multiplicative relationship departs most strongly from linearity. The raw residuals (the y-axis of this panel, in g/km) are strongly asymmetric, ranging from roughly, 700 g/km to about + 2,000 g/km, with the largest positive residuals concentrated at the highest fitted values, visual confirmation that the linear model under-predicts the largest CO₂ emission values.

*Normal Q–Q Plot:* The standardized residuals deviate from the reference line at both tails, most markedly in the upper tail, where the empirical quantiles rise well above the theoretical normal quantiles (and the lower tail falls below). This pattern indicates that the linear model systematically under-predicts the largest CO₂ emission values, which correspond to high-volume, low-speed scenarios where the multiplicative emission process is farthest from the linear approximation.

*Scale-Location Plot:* The LOWESS smoother displays a clear upward trend at the upper end (after an initial decline at very low fitted values), confirming that the magnitude of the standardized residuals is not constant across the range of fitted values. This heteroscedasticity signal is consistent with the U-shaped variance pattern observed in the Residuals vs. Fitted plot.

*Residuals vs. Leverage: *the standardized residuals extend from about −2 up to roughly +4, the large positive values again reflecting the under-predicted high-emission scenarios. Several observations fall near the Cook's distance contour of 0.5. These high-leverage points correspond predominantly to scenarios combining extreme traffic volumes with low speeds, the most congested operating conditions. Their presence signals that the coefficient estimates are sensitive to a relatively small number of extreme scenarios, an undesirable property for a model intended to represent emission behavior across the full range of freeway operating conditions.

In summary, the linear model for CO₂ exhibits heteroscedasticity, non-normal residuals, and influential observations. These violations are inherent to the additive functional form and are not remediable by removing individual observations; they reflect a structural mismatch between model form and data-generating process.

##### Quadratic model diagnostics (CO₂) (Fig. 5)

The quadratic specification augments the linear model with a squared speed term (S²), allowing the speed–emission relationship to exhibit curvature. The diagnostic panels occupy an intermediate position: while the quadratic model’s goodness-of-fit metrics improve upon the linear specification (Table 8), its diagnostic profile is inferior to the log-log model’s.

Residuals vs. Fitted: The residual dispersion is narrowest at low fitted values and widens substantially at higher fitted values, producing a funnel-shaped pattern. While the addition of the S² term captures curvature in the speed dimension, removing one source of systematic error, the quadratic model continues to operate in the original (untransformed) units of the dependent variable, where variance heterogeneity is inherent given the multiplicative emission structure. The consequence is that the residual spread at the upper end of the fitted range is notably larger than at the lower end, and the overall spread non-uniformity remains substantial, intermediate between the linear and log-log forms.

Normal Q–Q Plot: The Q–Q pattern is intermediate between the linear and logarithmic models. The central portion of the distribution follows the reference line reasonably well, but tail departures persist. The improvement over the linear model reflects the better fit to the speed dimension; the persistent deviations relative to the log-log model reflect the unresolved multiplicative structure in the traffic volume and emission relationship.

Scale-Location Plot: The LOWESS smoother exhibits an upward trend that is stronger than in the log-log model, confirming that the quadratic specification has not resolved the heteroscedasticity present in the untransformed data. The trend reflects the same structural issue as the linear model, albeit partially mitigated by the speed curvature term.

Residuals vs. Leverage: The pattern is broadly similar to the linear model. The quadratic term does not fundamentally alter the leverage structure because the most influential observations are determined primarily by extreme values of traffic volume, a variable that enters both the linear and quadratic models in identical (untransformed) form. Several observations approach the Cook's distance contour.

The diagnostic assessment of the quadratic model confirms that adding a squared speed term ameliorates curvature in the speed dimension but does not address the fundamental structural mismatch between the additive model form and the multiplicative process generating the emissions data. The quadratic model represents an improvement over the linear model, as the R² and AIC values in Table 8 attest, but it treats a symptom rather than the cause and is diagnostically inferior to the logarithmic specification.

##### Log-log model diagnostics (CO₂) (Fig. 6)

The logarithmic specification transforms both the dependent variable and the continuous predictors, yielding a model in which ln(CO₂ per km) is regressed on ln(traffic volume), ln(speed), trailer percentage, and strategy dummies. This transformation linearizes the multiplicative relationship governing emission generation. Among the three functional forms, the log-log model produces the best overall diagnostic profile.

*Residuals vs. Fitted:* The residuals on the log scale are scattered around the zero line with the most uniform spread of the three functional forms. The pronounced U-shape of the linear model is substantially reduced; a milder residual curvature remains visible in the LOWESS smoother (slightly elevated at both the low and high ends of the fitted range), but the dispersion is far more nearly constant than in either alternative specification. The mild curvature at the highest fitted values corresponds to the LBSL scenarios and the most congested conditions, where scenario-to-scenario variability is inherently greater. The standardized residuals are largely confined within ±2 standard deviations.

*Normal Q–Q Plot:* The standardized residuals adhere to the reference line more closely than in the linear or quadratic models. The central portion of the distribution (approximately the middle 80% of observations) follows the reference line closely. Minor deviations are observable in the extreme tails, but these are modest in magnitude. The overall pattern is consistent with approximate normality, an expected outcome when the logarithm of a product of multiple emission-influencing factors is modeled, following the central limit theorem.

*Scale-Location Plot: *The LOWESS smoother exhibits a flatter trend than in the linear or quadratic models, with only a mild residual U-shape; this indicates that the log transformation has been most effective at stabilizing the error variance. The remaining mild non-constancy is concentrated at both extremes of the fitted range, where the data density is lower. The homoscedasticity assumption is not perfectly satisfied, but the departure is substantially smaller than in either the linear or quadratic specifications, and standard inference based on OLS standard errors is likely to be reliable.

*Residuals vs. Leverage:* No observation exceeds the Cook's distance threshold of 0.5. All points fall within the region of acceptable influence, indicating that no single simulation scenario exerts disproportionate leverage on the coefficient estimates. This is a direct consequence of the log transformation, which compresses the scale of the traffic volume and emission variables, thereby distributing influence more evenly across the sample.

##### Synthesis and implications

**Fig. 5:**
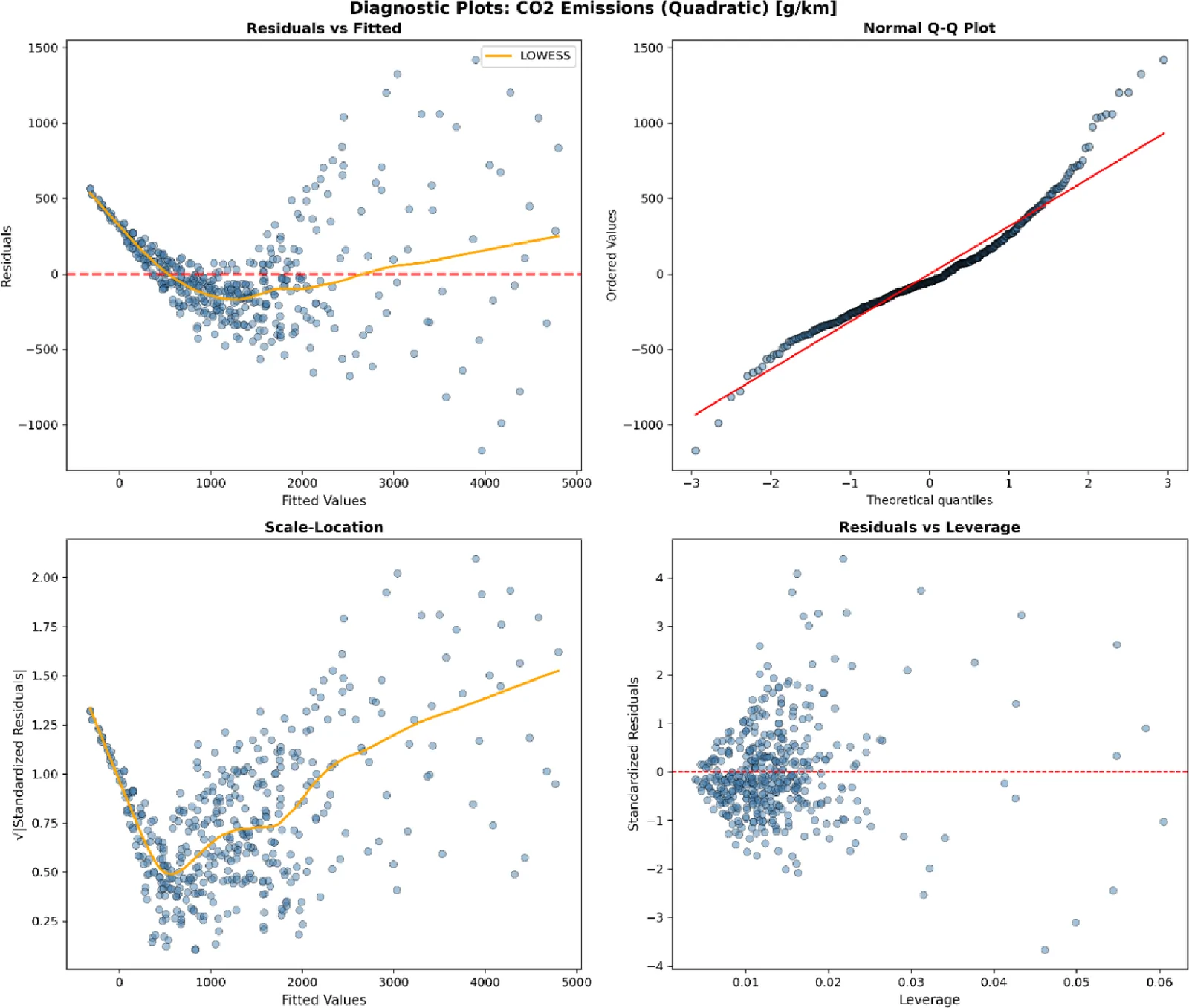
Diagnostic plots for the quadratic regression model fitted to CO₂ emissions per kilometer (g/km).

**Fig. 6:**
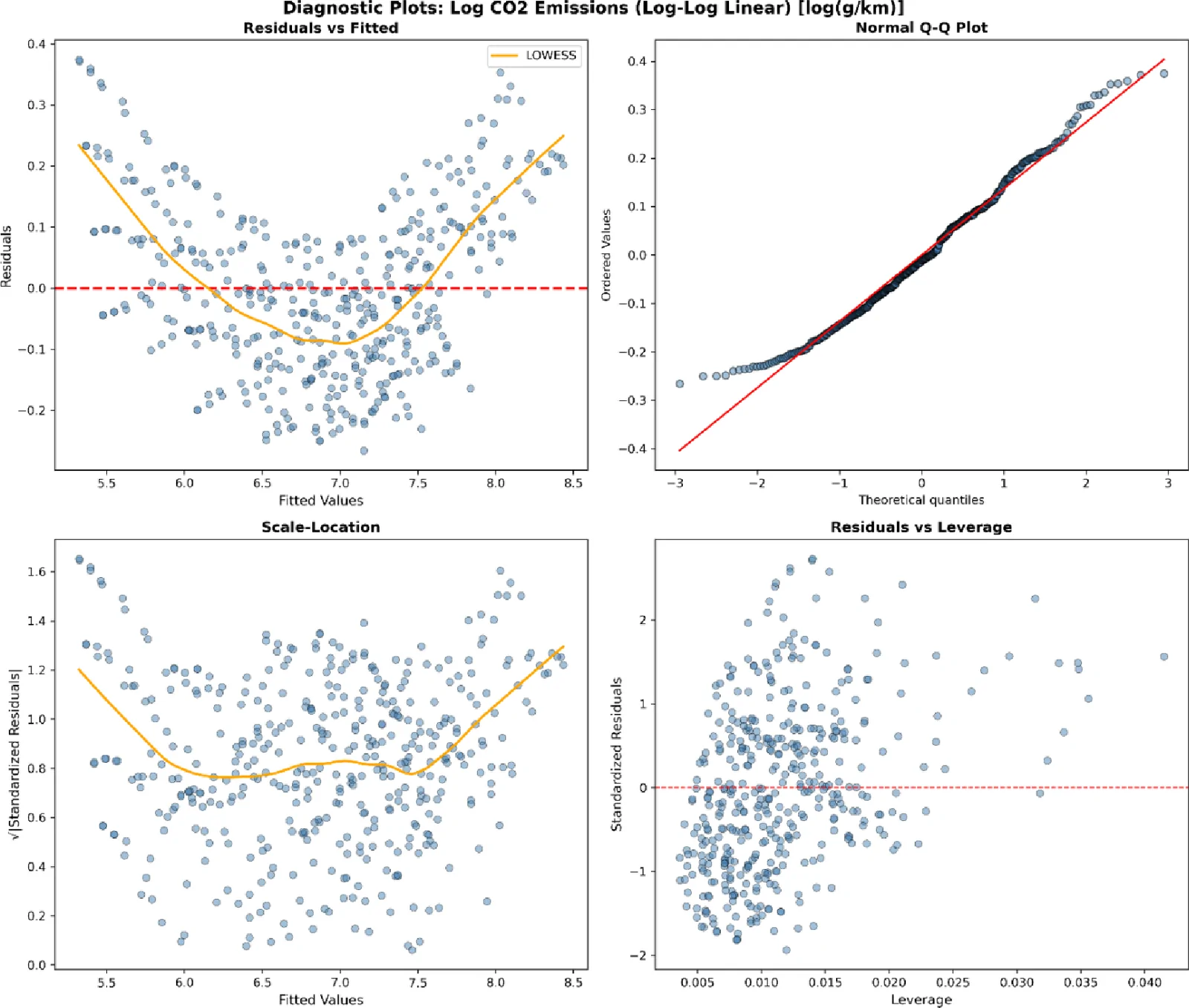
Diagnostic plots for the logarithmic (log-log) regression model fitted to CO₂ emissions per kilometer (g/km).

The diagnostic evidence presented in Figs. 4, 5 and 6 provides the visual justification for the quantitative model rankings in Table 8. The linear model (Fig. 4) is structurally mis-specified: its additive form cannot accommodate the multiplicative nature of emission generation, resulting in a U-shaped variance pattern, non-normal residuals, and sensitivity to influential observations. The quadratic model (Fig. 5) offers partial remediation by capturing speed curvature, its R² and AIC improve accordingly, but its diagnostic profile remains inferior to the log-log specification, with persistent heteroscedasticity and a funnel-shaped residual dispersion. The log-log model (Fig. 6), while not mathematically perfect, addresses the structural property of the data directly: by transforming to log space, it substantially linearizes the multiplicative emission process, produces the most uniform residual spread and the flattest Scale-Location trend among the three forms, yields approximately normal residuals, and eliminates influential observations.

The diagnostic assessment also validates the use of the log-log model’s coefficient estimates for inference and policy analysis, as discussed in the following section. The standard errors reported in Table 9 for the logarithmic specification are computed under OLS assumptions. While the log-log model does not satisfy these assumptions with mathematical perfection, the mild heteroscedasticity in the upper range of fitted values and minor tail deviations in the Q–Q plot warrant acknowledgment, the departures are sufficiently small that the hypothesis tests and confidence intervals are credible. By contrast, the more severe violations in the linear and quadratic models would require heteroscedasticity-robust standard errors and would still leave unresolved the structural implausibility of coefficient magnitudes estimated in the original units.

It is worth noting that the diagnostic patterns observed for CO₂ are representative of the full set of dependent variables. Across all six pollutants and fuel consumption, the diagnostic progression follows the same trajectory: systematic violations in the linear case, partial improvement with the quadratic model, and the best overall diagnostic profile under the log transformation. The consistent superiority of the logarithmic specification across the diagnostic suite, combined with its dominance on all quantitative goodness-of-fit criteria (Table 8) and the theoretical interpretability of its coefficients as elasticities, establishes the log-log functional form as the preferred specification for this study.

### Justification of the logarithmic functional form

The selection of the log-log model over the quadratic specification merits additional justification given the well-documented U-shaped relationship between speed and emissions^19,21^. The quadratic model, with its squared speed term, can indeed reproduce the U-shape across a wide speed spectrum (0–130 km/h). However, our experimental design focuses exclusively on freeway conditions with average speeds ranging from 70 to 115 km/h, a domain that lies predominantly to the right of the typical emission minimum for most pollutants. In this truncated domain, the empirical relationship between speed and emissions is monotonic: for CO_₂_, HC, NO_x_, PMx, and fuel consumption, emissions decline modestly with increasing speed (negative elasticities in Table 9), reflecting the dominance of reduced congestion and smoother traffic flow over the aerodynamic drag penalty at these speeds. CO is the sole exception, exhibiting a modest positive elasticity (+ 0.29).

The log-log model offers three distinct advantages for this study’s policy evaluation objectives:

1. *Interpretability:* The coefficients are direct elasticities, quantifying the percentage change in emissions per 1% change in traffic or speed. This metric is directly applicable to marginal policy analysis.
2. *Domain appropriateness:* Within the observed speed range, the relationship is log-linear, as evidenced by the higher adjusted R² and lower information criteria relative to the quadratic form (Table 8).
3. *Prediction robustness:* As demonstrated in Eqs. 4–6, the quadratic model can extrapolate to negative emission values outside the calibration range, whereas the log model guarantees positive predictions, a critical property for out-of-sample policy forecasting.

Consequently, while we acknowledge global U-shape, the log-log specification is the preferred tool for the comparative elasticity analysis that forms the core contribution of this work.

### Elasticity analysis

*Traffic elasticity:* Traffic elasticity results indicate that most emissions and fuel use respond elastically to traffic volume, with elasticity values greater than 1. This implies that a 1% rise in traffic volume is associated with an increase of more than 1% in emissions, as shown in (Table 11). PMx was close to unitary (1.04), making it the least elastic of the six outcomes; all remaining pollutants and fuel exhibited elasticities between 1.47 and 1.60, indicating a stronger-than-proportional response to traffic volume increases. This indicates that rising traffic volumes are the main reason emissions are increasing.

**Table 11:** Traffic volume elasticities of emissions and fuel consumption.

Emission/fuel	Traffic elasticity ($\:{\beta\:}_{ln\left(V\right)}$)	% Change per 1% ↑ traffic volume
CO	1.60	+ 1.60% CO emissions
CO_₂_	1.47	+ 1.47% CO_₂_ emissions
HC	1.48	+ 1.48% HC emissions
NO_x_	1.48	+ 1.48% NO_x_ emissions
PMx	1.04	+ 1.04% PMx emissions
Fuel	1.48	+ 1.48% fuel consumption

*Speed elasticity: *Most pollutants and fuel showed a negative inelastic relationship with speed (Table 12). A 1% increase in average speed reduced CO₂ and fuel consumption by 0.54%, and NOx by 0.71%. CO was the exception, exhibiting a small positive elasticity (+ 0.29%), indicating it increases slightly with speed.

**Table 12:** Speed elasticities of emissions and fuel consumption.

Emission/fuel	Speed elasticity ($\:{\beta\:}_{ln\left(S\right)}$)	% Change per 1% ↑ Speed
CO	+ 0.29	+ 0.29% CO emissions
CO₂	−0.54	− 0.54% CO₂ emissions
HC	−0.16	− 0.16% HC emissions
NO_x_	−0.71	− 0.71% NO_x_ emissions
PMx	−0.06	− 0.06% PMx emissions
Fuel	−0.54	− 0.54% fuel consumption

It is important to distinguish between statistically significant and non-significant strategy effects. The USL strategy was initially estimated in a full model containing both LBSL and USL dummy variables. For CO₂, HC, and fuel consumption, the USL coefficient was not statistically significant at α = 0.05 accordingly, USL was excluded and these three models were re-estimated without the USL variable (refer to Table 9).The reported LBSL coefficients for CO₂, HC, and Fuel in Table 14 therefore reflect the reduced model estimates. For CO, NO_x_, and PMx, both USL and LBSL were retained in the final model as both were statistically significant.

In the final models, USL significantly reduced CO emissions by 7.6% relative to DSL, while both USL and LBSL increased NO_x_ and PMx relative to DSL, meaning DSL is the lower-emission strategy for NO_x_ and PMx. Specifically, USL increased NOx by 14.7% and PMx by 4.6% relative to DSL. LBSL differed significantly from DSL on all six outcomes. For CO₂, HC, and Fuel, only LBSL differed significantly from DSL; USL did not differ significantly from DSL on these three outcomes and was excluded from the re-estimated models.

*Heavy vehicle impact:*The proportion of heavy vehicles (trailers) significantly increased emissions for all pollutants except CO (Table 13). NOx was most sensitive, increasing by 13.6% for every 1%-point increase in heavy vehicle share, highlighting the disproportionate impact of diesel truck operations.

**Table 13:** Effect of heavy vehicle percentage on emissions and fuel (semi-elasticity).

Emission/fuel	Coefficient ($\:{\beta\:}_{T}$)	% Change per 1% ↑ trailer
CO	−0.0042	−0.41%
CO₂	+ 0.0278	+ 2.82%
HC	+ 0.0415	+ 4.24%
NO_x_	+ 0.128	+ 13.66%
PMx	+ 0.0431	+ 4.40%
Fuel	+ 0.0276	+ 2.79%

### Speed limit strategy comparison

The core findings that compare the environmental performance of LBSL and USL against the baseline DSL strategy are shown in (Table 14).

**Table 14:** Percentage change in emissions and fuel for LBSL and USL relative to DSL.

Emission/fuel	LBSL Coeff.	LBSL vs. DSL (%)	95% CI	USL Coeff.	USL vs. DSL (%)	95% CI
CO	+ 0.2136	+ 23.8%	(+ 19.0% to + 28.9%)	−0.0795	−7.6%	(− 11.1% to − 4.0%)
CO₂	+ 0.2407	+ 27.2%	(+ 22.3% to + 32.3%)	**—** ^a1^	**—** ^a2^	**—** ^a3^
HC	+ 0.2171	+ 24.2%	(+ 19.5% to + 29.2%)	**—** ^b1^	**—** ^b2^	**—** ^b3^
NO _x_	+ 0.3839	+ 46.8%	(+ 33.9% to + 60.9%)	+ 0.1375	+ 14.7%	(+ 5.1% to + 25.2%)
PMx	+ 0.0442	+ 4.5%	(+ 2.3% to + 6.8%)	+ 0.0451	+ 4.6%	(+ 2.5% to + 6.8%)
Fuel	+ 0.2404	+ 27.2%	(+ 22.3% to + 32.3%)	**—** ^c1^	**—** ^c2^	**—** ^c3^

— : USL was not statistically significant at α = 0.05 for these outcomes. The model was re-estimated without USL for CO₂, HC, and Fuel; accordingly, no USL coefficient is reported for these three outcomes (refer to Table 9). The LBSL coefficients for CO₂, HC, and Fuel reflect the reduced model estimates. All remaining coefficients are from models retaining both LBSL and USL.For the full log-log model: (1) for CO2, ^a1^: +0.0155, ^a2^:+1.60%, and ^a3^:(−1.7% to +4.9%); (2) for HC, ^b1^: −0.0312, ^b2^: −3.1%, and ^b3^: (−6.6% to +0.6%); and (3) for Fuel, ^c1^: +0.0151, ^c2^: +1.5%, and ^c3^: (−1.7% to +4.9%)..

*Lane-based speed limits (LBSL):* This strategy was associated with poorer environmental performance across every metric assessed. NO_x_ emissions increased by 46.8% and CO₂ emissions/fuel consumption by about 27% compared to DSL. The LBSL emissions penalty arises from two mechanisms. First, LBSL constrains vehicles to operate in lower-speed lanes (inner and middle lanes at 80–100 km/h), where the HBEFA speed-emission curve shows elevated emission rates for CO, HC, and CO₂ relative to the emission minimum, which for most pollutants occurs below the 70 km/h lower boundary of the study’s operational speed range, as discussed in Sect. "Justification of the logarithmic functional form". Second, although LBSL reduces total lane-change frequency by 15–61% relative to USL (using the same simulation framework from our previous work)^29^, the transitions that do occur involve larger speed differentials between adjacent lanes (up to 40 km/h), requiring greater deceleration upon entering a slower lane and subsequent re-acceleration to resume travel speed, generating emission spikes. This interpretation is consistent with the higher standard deviation of delay under LBSL (Table 5) and the elevated NO_x_ emissions, which are particularly sensitive to transient high-acceleration events.

*Uniform speed limits (USL):*This strategy produced mixed results. It reduced CO emissions by 7.6%, but NO_x_ and PMx emissions increased by 14.7% and 4.6%, respectively. The effects on CO_₂_, HC, and fuel use did not differ statistically from those of DSL; USL was therefore excluded from the re-estimated reduced models for these three outcomes, and no USL coefficient is reported for CO₂, HC, or Fuel in Table 13. The absence of a significant USL effect on CO₂, HC, and Fuel is itself a substantive finding, indicating that USL and DSL produce statistically equivalent outcomes for these metrics under the tested condition. A uniform speed cap can require heavy vehicles to travel below their optimal operating speed, which may increase NO_x_ and PMx emissions.

The negative CO coefficient for the trailer-share variable (Table 9) requires a mechanistic explanation. CO emissions from gasoline engines are dominated by incomplete combustion during transient acceleration events. A higher share of heavy vehicles smooths traffic flow by reducing acceleration capability, decreasing lane-change frequency, and lowering speed variance, which in turn reduces the frequency and magnitude of transient acceleration events that produce CO. This effect is statistically significant (*p* < 0.001) in the log-log model. We present this as a plausible interpretation rather than a confirmed mechanism; the other heavy-vehicle effects (CO₂, fuel, NOₓ, PMx) have the expected positive sign, consistent with the higher absolute emission rates of heavy-duty diesel engines.

It is worth noting that while the global speed-emission relationship is U-shaped, the operational speed range on high-speed highways typically lies to the right of the minimum-emission speed. In this study, the observed average speeds across all scenarios ranged from approximately 70 km/h to 115 km/h. Within this restricted domain, the emission function is monotonically increasing or decreasing depending on pollutant type and congestion level. For the purpose of estimating policy-relevant elasticities, which measure proportional changes at the margin, a log-log specification is both theoretically appropriate and empirically well-fitting, as demonstrated by the superior model diagnostics presented in Sect. 3. The quadratic specification, while capable of capturing the global U-shape, yields elasticity estimates that vary with speed and does not provide a single, interpretable marginal effect suitable for policy comparison across scenarios.

The log-log model’s superiority is further supported by residual diagnostics (refer to Figs. 4, 5 and 6): the Scale-Location (standardized-residual versus fitted values) plot shows approximately constant variance for the log model, whereas the linear model exhibits heteroscedasticity in both the Residuals vs. Fitted and Scale-Location plots; the Q-Q plot confirms that the log model residuals are closer to normal. Additionally, the log model yields realistic positive predictions in out-of-sample scenarios (Eqs. 4–6), whereas the quadratic model can produce negative values.

### Policy implications and recommendations

Based on the multi-pollutant analysis, the recommended strategy depends on the primary policy objective, as shown in Table 15. For an overall balanced environmental performance, DSL shows the most favorable relative performance across the 432 tested scenarios. It minimizes NO_x_ and PMx, critical pollutants from heavy vehicles, without significantly worsening CO₂, HC, or fuel consumption compared to USL, and it vastly outperforms LBSL. However, these findings are conditional on the corridor geometry, the demand range, the trailer share range, and the emission factors used in the simulation, and should not be generalized to other contexts without re-evaluation.

**Table 15:** Recommended speed limit strategy under the tested conditions, by environmental objective.

Objective	Recommended Strategy	Rationale
Minimize CO	USL	7.6% reduction vs. DSL
Minimize CO₂	DSL or USL	LBSL significantly worse
Minimize HC	DSL or USL	LBSL significantly worse
Minimize NO_x_	DSL	Both USL and LBSL alternatives increase NO_x_
Minimize PMx	DSL	Both USL and LBSL alternatives increase PMx
Minimize Fuel	DSL or USL	LBSL significantly worse
Overall Balance	DSL	Best overall performance

### Implications for policy in heterogeneous fleet contexts

Although the simulation parameters and emission factors were derived from HBEFA and do not reflect the specific fleet composition of any single developing nation, the relative performance of the three strategies offers transferable insights for jurisdictions characterized by mixed traffic and aging diesel fleets.

- *Differential Speed Limits (DSL):* The strategy comparison results, summarized in Tables 13 and 15, show that DSL achieves the best overall environmental performance across the 432 tested scenarios. It is the preferred strategy for minimizing NO_x_ and PMx, the pollutants most strongly associated with heavy vehicle operations, while producing CO₂, HC, and Fuel outcomes that are statistically equivalent to USL. These advantages are most relevant for jurisdictions with high shares of heavy vehicles, where NO_x_ and PMx emissions represent the dominant environmental concern. The findings are conditional on the specific corridor geometry, demand range, trailer share range, and emission factors used in the simulation; they should not be extended to other settings without further evaluation using locally calibrated parameters.
- *Lane-Based Speed Limits (LBSL):* The substantial emission penalties associated with LBSL, particularly for No_x_, suggest that its operational benefits (e.g., reduced speed variance, improved lane discipline) must be weighed carefully against environmental disbenefits. If LBSL is considered for implementation, it should be accompanied by rigorous before-after emission monitoring.
- *Uniform Speed Limits (USL):* USL offers a modest reduction in CO but at the expense of increased NO_x_ and PMx. This trade-off implies that USL may be most appropriate in contexts where CO is the pollutant of primary concern (e.g., urbanized corridors with high localized CO hotspots) and where NO_x_ and PMx can be managed through other policy instruments.

We caution that direct translation of the numerical elasticities reported herein to a specific country’s policy requires local calibration of vehicle fleet composition and emission factors. The qualitative ranking of strategies (DSL preferred over LBSL; USL as a mixed case) is, however, expected to hold across a range of fleet assumptions given the underlying physical mechanisms of speed variance and heavy vehicle dynamics.

Although the simulation parameters and emission factors were derived from HBEFA and reflect European fleet characteristics, including diesel fleet age, fuel quality, and exhaust after-treatment standards that differ substantially from Egyptian conditions, the relative performance of the three strategies offers transferable insights. This is because the fleet composition is held constant across all strategies, so the strategy ranking depends on speed-acceleration patterns rather than absolute emission factors. However, the absolute emission values should not be transferred to other contexts without re-parameterization using locally derived emission factors.

The following dimensions limit the transferability of the present findings to other contexts:

1. *Corridor geometry:* The study uses a single 3.6 km, 3-lane highway segment with one weaving section. Generalization to longer corridors, multi-segment corridors with multiple interchanges, or different weaving configurations is not directly supported by the present results.
2. *Calibration framework:* The Wiedemann-99 parameters are adapted from^26,29,36^. Generalization to other driving cultures would require re-calibration.
3. *Simulation tool.* The study uses SUMO with HBEFA4 emission classes. Other microsimulation tools (VISSIM, Paramics, CORSIM, etc.) may produce different absolute values, although relative strategy rankings are expected to be more robust to tool choice.
4. *Emission factors:* HBEFA factors are derived from European driving cycles and fleet composition. Absolute emission values would differ for North American (MOVES) or other regional factor sets. Relative strategy comparisons, which are the focus of the present study, are expected to be more robust.
5. *Dynamic speed limits:* The study considers only static speed limit strategies. Variable speed limits (VSL) and dynamic speed limit systems, which are increasingly used in practice, are outside the scope of this study.

## Conclusion, limitations, and future work

This study performed comprehensive microsimulation to assess three highway speed limit strategies. The log-log regression model gave the most reliable results for prediction and for estimating elasticity. For an overall balanced environmental performance, DSL shows the most favorable relative performance across the 432 tested scenarios. It minimizes NO_x_ and PMx, critical pollutants from heavy vehicles, without significantly worsening CO₂, HC, or fuel consumption compared to USL, and it outperforms LBSL. However, these findings are conditional on the corridor geometry, the demand range, the trailer share range, and the emission factors used in the simulation, and should not be generalized to other contexts without re-evaluation.

The Lane-Based Speed Limit (LBSL) strategy leads to higher emissions overall. In contrast, the Uniform Speed Limit (USL) strategy involves a trade-off: it lowers CO emissions but raises the levels of other pollutants, specifically NO_x_ (+ 14.7%) and PMx (+ 4.6%) relative to DSL, while producing statistically equivalent outcomes to DSL for CO₂, HC, and Fuel. Effective environmental policy should account for differences in fleet composition, and multi-pollutant impacts rather than adopting a one-size-fits-all speed management approach.

This study adds to existing research and informs practice in the following ways:

- The study methodological contribution is a structured comparison of linear, quadratic, and log-log specifications for predicting emissions. The results show that the log-log form fits the data better than the alternatives and is well-suited for estimating elasticities. The study also offers a clear framework for multi-pollutant, multi-scenario emission analysis based on microsimulation data.
- The empirical work reports three main results: it measures how the strategy changes six indicators of emissions and fuel use, estimates how traffic volume and vehicle speed respond to changes in conditions and reports uncertainty bounds for those estimates, and examines how the proportion of heavy vehicles shape emission results, showing that fleet composition matters for emissions.
- The practical outputs of this work include policy scenario analyses that compare alternative strategies, estimates of the benefits from reducing heavy vehicle traffic, and decision-support tables that transportation agencies can use when setting speed limits and managing traffic.

This study also has several limitations:

- These results are based on simulated driver behavior and may not reflect real-world variation in compliance or in how enforcement is applied.
- The Wiedemann-99 car-following parameters were adopted from Saccomanno et al.^36^, who calibrated them against NGSIM U.S. freeway trajectory data, and from Shahdah et al.^26,29^, who recommended CC0 to reflect current Egyptian driving behavior. The parameters were not re-calibrated against Egyptian highway trajectory data. The transfer of NGSIM-calibrated parameters to Egyptian conditions is a recognized limitation. As such, the absolute emission values should not be interpreted as real-world predictions for any particular corridor^44,45^.
- The transferability limitations related to HBEFA emission factors and European fleet characteristics are discussed in detail in Sect. "Implications for Policy in Heterogeneous Fleet Contexts". In summary, while relative strategy comparisons are expected to be robust under consistent fleet assumptions, absolute emission values should not be transferred to other contexts without re-parameterization using locally derived emission factors.
- The 3.6 km corridor length, while sufficient for capturing the dynamics of a single weaving segment and basic freeway section, limits the generalizability of the findings to longer corridors where speed harmonization effects may differ. Future work should extend the analysis to network-level evaluations.
- The study did not assess dynamic speed limits or how different speed strategies work with active traffic management tools such as variable message signs.

Additionally, Future work should integrate locally calibrated car-following parameters from instrumented Egyptian highway data to address the parameter transfer limitation identified in this study. It should verify the simulation results against real traffic and emissions data from highways that run under different speed ranges. It should also examine how dynamic speed limit systems, which respond to real-time traffic and weather conditions, affect the environment. Finally, future work should broaden the analysis to cover life-cycle emissions from vehicle fleets and assess how newer electric and hybrid vehicles may change the overall impact of the strategy.

## Data Availability

The code and dataset generated is available from the corresponding author upon reasonable request.
